# Labour-Efficient In Vitro Lymphocyte Population Tracking and Fate Prediction Using Automation and Manual Review

**DOI:** 10.1371/journal.pone.0083251

**Published:** 2014-01-03

**Authors:** Rajib Chakravorty, David Rawlinson, Alan Zhang, John Markham, Mark R. Dowling, Cameron Wellard, Jie H. S. Zhou, Philip D. Hodgkin

**Affiliations:** 1 National ICT Australia, Dept. of Electrical & Electronic Engineering, the University of Melbourne, Melbourne, Victoria, Australia; 2 Walter and Eliza Hall Institute of Medical Research, Parkville, Melbourne, Victoria, Australia; 3 Department of Medical Biology, the University of Melbourne, Melbourne, Victoria, Australia; Georg Speyer Haus, Germany

## Abstract

Interest in cell heterogeneity and differentiation has recently led to increased use of time-lapse microscopy. Previous studies have shown that cell fate may be determined well in advance of the event. We used a mixture of automation and manual review of time-lapse live cell imaging to track the positions, contours, divisions, deaths and lineage of 44 B-lymphocyte founders and their 631 progeny *in vitro* over a period of 108 hours. Using this data to train a Support Vector Machine classifier, we were retrospectively able to predict the fates of individual lymphocytes with more than 90% accuracy, using only time-lapse imaging captured prior to mitosis or death of 90% of all cells. The motivation for this paper is to explore the impact of labour-efficient assistive software tools that allow larger and more ambitious live-cell time-lapse microscopy studies. After training on this data, we show that machine learning methods can be used for realtime prediction of individual cell fates. These techniques could lead to realtime cell culture segregation for purposes such as phenotype screening. We were able to produce a large volume of data with less effort than previously reported, due to the image processing, computer vision, tracking and human-computer interaction tools used. We describe the workflow of the software-assisted experiments and the graphical interfaces that were needed. To validate our results we used our methods to reproduce a variety of published data about lymphocyte populations and behaviour. We also make all our data publicly available, including a large quantity of lymphocyte spatio-temporal dynamics and related lineage information.

## Introduction

### 1.1 Motivation

The motivation for this paper was to explore the impact of semi-autonomous (assistive) software interfaces on the productivity and quality of live-cell imaging studies. With these questions in mind, this paper describes our efforts to develop software tools for cell tracking and lineage modelling (also known as genealogical reconstruction), specifically *in vitro* analysis of B-lymphocytes. We focus on the interfaces and human-computer interaction necessary to bridge the gap between convenient but inaccurate automatic tracking, and more accurate but time-consuming manual work. To measure success against these objectives, we try to fulfil three objectives: Efficiency, validity and utility. Efficiency captures the objective that the software should produce results within a short period of time using less effort than existing methods. Validity is an attempt to measure whether the results produced are accurate enough. Utility explores whether the type and qualities of data produced using these methods is useful and interesting.

### 1.2 Contributions

To evaluate this software and these methods, we studied small populations of lymphocytes *in vitro* over several generations. We tracked a total of 675 cells for up to 7 generations, over 1296 frames and 108 hours.

Results from these experiments support our claims of accuracy and efficiency, and in the process we have produced an unprecedented quantity of new data about changes in lymphocyte size and motility over generations. The tracking data has been made available in raw form for further study, including details not analysed here such as cell contours. We have made some novel observations from these data, primarily because we provide a combined model of lymphocyte lineage, generation, fate, frame-by-frame segmentation, contours and tracking for a large quantity of cells. The software we used to produce these data is called TrackAssist. Full source code has been released under an open-source licence.

A key contribution of this paper is to demonstrate the impact of the rich data captured by these methods. As an example, we show that it is possible to predict lymphocyte fates before they occur, with good accuracy, by segmenting and tracking cells in time-lapse imaging. After training on the semi-automated cell tracking data, a fully-automated machine learning method was able to predict more than 90% of individual cell fates using only imaging data captured during a window of time prior to 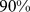 of cell fate outcomes. This raises the possibility of realtime intervention to segregate or treat cells according to phenotype or fate [1], or other potential applications including high content screening [2]–[4]. With recent advances in cell segmentation, these methods could be generalized to other cell types.

To demonstrate validity, we have used our methods to reproduce all the graphical results given in [5], albeit with a mouse genetically modified so that all cells produce GFP and with different illumination conditions. We found that our results agreed closely with existing data with the exception of some low frequency events not previously observed. These were all investigated and found to represent correct reports of observable phenomena, discussed later in this paper. We do not believe that these observations refute any previous results, rather they demonstrate that this new approach can yield extra information compared to lower-volume fully manual annotation processes.

To demonstrate efficiency, we present a greatly increased volume of results compared to earlier work. This is especially true of the spatio-temporal dynamics of lymphocytes, which was limited to progeny of 9 founder cells in the original work. We also report the human-hours of labour required to produce these results, which we compare to anecdotal reports of effort required to produce similar results with popular methods.

A primary contribution is insight into how interaction and automation can be used effectively for *in vitro* time-lapse cell tracking. This topic is of wider applicability than lymphocyte analysis, but application of combined automation and manual review methods has not been thoroughly described in cell biology. In practice, the gap between automation and manual cell tracking is commonly encountered but methods of bridging it are not well documented. Many research groups develop, download or purchase suites of tools for cell tracking, but rarely publish their experiences using these tools.

### 1.3 Background

A short review of relevant methods and software is given below. An alternative thorough review dated 2012 can be found in Meijering et al [6]. Meijering et al discuss the recent proliferation of publications about cell tracking, methods of cell detection and tracking, a review of available software tools and (briefly), lineage reconstruction, all topics of relevance to this paper.

#### 1.3.1 Efficient and accurate image interpretation

Time-lapse microscopy collects a great quantity of detailed information but in a form that is not easy to draw inference from. To generate statistics about cells and cell populations, the images must be interpreted. Manual image interpretation is typically perceived as time-consuming but a “gold standard” for accuracy. The effort involved reduces the volume and hence statistical power of results. Automated image interpretation is more consistent, but is still prone to systematic bias. For example, a method of detecting cells might systematically ignore smaller cells. This is a serious issue if these biases can affect the hypotheses being considered. It is often possible to automate image interpretation tasks with high accuracy, but it is usually very difficult to approach human performance under difficult conditions. Diminishing returns from additional algorithm improvements can be expected as with any complex system.

Experimental evidence shows that human image interpretation is less accurate, more optimistic and more biased than commonly believed. For example, Bruce et al showed that human face-recognition performance is worse than expected [7], and that humans often fail to recognise faces in typical CCTV images [8]. Although automated methods will also be biased, their bias is consistent and therefore its impact can be minimized or excluded. With a multitude of human interpreters, this may not be possible. For example, Abraham et al [9] show some strong and unexpected biases in clinical interpretation of cataract images. Reviewers differ in their biases, with all reviewers having more bias than automation for less severe cases. The thesis of Fendley [10] gives a good overview of the types and causes of bias in human image interpretation which may help researchers to develop mitigating procedures. In particular, she discusses the impact of fatigue and repetition which may affect in cell tracking research due to the volume of cells involved. Radiology is another clinical image interpretation purpose where bias must be minimized. Sica [11] gives a thorough overview of sources and mitigating strategies in Radiology. The issue of image interpretation bias has not been thoroughly discussed in cell biology, but use of software tools that minimize tedious and repetitive labour can be expected to reduce human errors in this domain like any other.

When tracking cells, a number of factors demand very high accuracy. It is relatively costly to perform experiments and capture images, preventing us from using large data volumes to increase confidence. Typically, several days are required to conduct a lymphocyte lineage experiment. Since we wish to model cell lineage, the events we must observe are not independent and not instantaneous. It is necessary to track individual cells accurately for generations (approximately 100 hours) to create models of lineage. Although our lymphocytes have some varying observable features (size and fluorescence intensity), these features are ambiguous and not constant. If cells do not have visible characteristics that uniquely identify them, we must exploit positional information that is confounded unless we identify them regularly (e.g. every few minutes in the case of highly mobile lymphocytes). This means that to build lineage models, we must correctly associate every cell over hundreds of observations to form tracks. This type of cell tracking problem occurs frequently. Although our goal is tracking for lineage reconstruction, other reasons for long term cell tracking are modelling cell migration patterns [12], [13], chemotaxis [14], [15], and understanding the behaviour of parasites, bacteria [16], and other cells [17] where motility is critical to their function. Researchers already use a variety of software tools to assist with time-lapse microscopy image interpretation and cell tracking. Most current tools fall into two categories, which we will name “automatic” and “manual”. Although many tools have both automatic and manual options, none have good support for use of both options interchangeably.

#### 1.3.2 Manual cell tracking software

We describe software as “manual” if it features interfaces that allow the user to edit detections and create or delete tracks. Examples include Fiji's manual tracking plugin [18], Tim's Tracking Tool (TTT) [19]–[21] and some tools within Metamorph [22]. In cell tracking, there are two main tasks: Detection (segmentation in images), followed by association. The association step is often implemented by requiring the user to follow a cell lineage through an entire sequence, clicking on the cell in each frame. This is repeated for other lineages or individual cells within a lineage. The method is efficient because it is usually easy to track a single cell, and because the current lineage is implicit therefore only one interaction is needed per frame (e.g. one click). Some tools, such as TTT, explicitly handle cell divisions and are able to associate progeny with parent cells. Moogk [23] describes a Matlab tool that was used to track lymphocytes and build lineage trees, but does not extract cell contours. Tools focusing on tracking tend not to segment cells for the user, but only record the position clicked. The “track objects” plugin for Metamorph does exactly this.

TTT and Moogk explicitly encode tracking loss and cell deaths separately. This is a useful feature because lost tracks can be excluded from results where necessary (avoiding censorship effects), and the distinction can easily be made when manually tracking cells. In immunology, correlations between related cells' death times help to explain population changes [24], [25]. However, details of these relationships are more easily and accurately defined as the number of observed cells increases.

Tracking tools based on image processing applications, such as CellProfiler [26], Fiji and Metamorph, tend to provide good facilities for automatic segmentation and allow the user to associate cell detections between frames by clicking anywhere in an automatically-segmented cell. Segmentation by itself is a useful application, having a range of uses including cell counting in high content experiments. CellTracker [27] has sophisticated cell segmentation with smart tools for manual contour correction. This improves spatial data output and is quicker because the user can be less careful about definitions of cell position, which may be poorly defined for irregularly shaped cells. The results from these programs tend to include cell contours and good centroid metrics. Both Fiji and Metamorph have plugins that allow the user to follow individual cells throughout image sequences. Fiji and metamorph also have smart “manual” tools for improving automatic cell segmentation. These tools avoid the user having the laborious task of manually drawing the contour by fixing over and under-segmentation errors with “hints” from the user. For example, the user can choose to merge selected contours, or automatically cut cells falsely joined together using smart rules (e.g. cut along the thinnest or darkest line).

#### 1.3.3 Automated cell tracking software

Many tools provide automatic cell tracking with reasonable success, despite generally using only cell positional information and Brownian motion models to associate detections. Critically, researchers are experienced with the limitations of automatic segmentation and tracking software and try to design their imaging experiments to match the capabilities of the software. Volocity [28] Metamorph [22], CellTrack [29], CellProfiler [26] and CellTracker [27] all have flexible and powerful automatic tracking tools, mostly with automatic segmentation methods as well. However, while these tracking tools are all sufficient for some purposes none provides good interfaces for manual track review and correction where very high accuracy is needed.

Instead, tracks are typically exported as text files for manipulation in third-party tools. Once exported, it is difficult to associate amended track details with the original images and therefore manual correction is very difficult. Even tools such as Metamorph with both manual and automatic tracking features do not have the ability to share data between manual and automatic tracking systems. In Volocity, for example, automatic tracking results can only be improved by repeating the automatic process with varying parameters. The exception is Imaris [30], a commercial product which does have a graphical user interface for track editing.

#### 1.3.4 Lineage modelling software

As cell imaging methods and tracking capabilities have improved, lineage modelling software has become available. A synonym of lineage modelling is genealogical reconstruction. LineageTracker [31] extends ImageJ and FIJI adding manual lineage modelling. It was written by the Warwick Systems Biology Centre. The example use of LineageTracker is modelling populations of constantly fluorescing mouse stem cells, in which initial segmentation is automatic, tracking is automatic or manual (depending on plugin) and lineage modelling is automatic. Manual editing or correction of lineages is not supported by a graphical interface - instead track IDs are entered into a table. Regarding the state of the art in lineage reconstruction, Downey et al [32] comment: “Lineage tracking is an emerging field and there is no commercial software available”… “fully automated reconstruction of cell lineages in experiments with low temporal resolution are currently not within reach”. Kang Li [33] et al describe an attempt and results from fully automatic lineage reconstruction without manual correction.

A group at CMU is trying to develop a fully-automatic web based cell tracking system, allowing users to submit images and later download frame-by-frame segmentation, tracking, and lineage reconstructions [34]. They do have interfaces for evaluating their automation, which are written in Matlab. However, they have not yet reported good accuracy with fully automated methods.

#### 1.3.5 Alternatives to inter-generational tracking

There are alternatives to long term live cell imaging and tracking, but they are not feasible in all circumstances especially where continuity of observation is needed. The most common are flow cytometry [15], [35] and high-content screening, that are effectively taking random samples from various cell populations at time intervals. If imaging is used, samples of cell properties such as motility [6], size and contour can be measured. One benefit of flow cytometry is that it also enables *ex vivo* experiments, meaning that conditions are more realistic than the *in vitro* environments used for continuous tracking. Another benefit is that results are inherently more robust due to large sample sizes. These techniques also allow sampled cells to be divided into categories, for example by Fluorescence-activated cell sorting (FACS) [36] or by Dielectrophoresis (DEP). Some of the information gained from cell tracking can be inferred from observable variations in sampled cell populations, but other questions cannot be answered by population sampling. For example, the relationships between sibling lymphocytes discovered by [37] could not have been discovered by population sampling, because e.g. division time correlations could equally have been explained by other mechanisms. However, once known, models derived from time-lapse microscopy can be applied to the interpretation of population samples and verified *ex vivo*. Besides immunology, pedigree or lineage is also important for modelling stem cells [19]. In summary, time-lapse microscopy is necessary when the development of cell morphology, motility, time-dependent and familial correlations or any feature requiring preservation of identity is of interest.

Zilman et al [25] give a good discussion of the application of intracellular labelling and multi-channel flow cytometry to measurement of population structure of proliferating and dying lymphocytes over several generations. One of the topics covered is whether correlations in division times between daughter and mother cells can be inferred from the cytometry division data, concluding “These results indicate that in some cases, even if data have intrinsic correlations between generation times of mothers and daughters, model without such correlations can describe the data well. Furthermore, it might be difficult to obtain unambiguous inference based on the cell division data alone.” [25]

### 1.4 Our approach

#### 1.4.1 Objectives

We hoped to demonstrate whether labour-efficient, “assistive” software tools could produce new insights into cell behaviour, by enabling the production of larger and more detailed *in vitro* cell tracking data.

“Assistive” means that some automation is used, but augmented with good tools for manual review and correction. We are not the first to consider the importance of good manual review and correction tools for cell tracking studies. Rapoport et al [38] recently published a very large study of pancreatic stem cell tracking using time-lapse microscopy. To improve accuracy over fully autonomous methods they developed interfaces for manual review, and automatic methods of detecting likely errors to be reviewed. The automation and interfaces thereby support highly efficient and accurate manual work. Our approach is very similar, but with assistive tools and manual review of both segmentation and tracking. Another example of interactive software developed to support manual review of tracking is Scherf et al [39].

We expect the assistive approach would also work for other image interpretation and tracking problems, where high accuracy is demanded and automation is difficult.

#### 1.4.2 Key design considerations

To evaluate our approach we developed and evaluated a combination of automatic and manual cell segmentation and tracking tools in a common graphical interface. We considered writing plugins for existing software (such as ImageJ [40]), but decided this approach did not provide enough freedom to configure graphical user interfaces. Given our objectives, this was a serious concern.

Although we have released our software under an open-source licence, we expect that others will benefit from our descriptions of key software features, as much as from the software itself. Some of the key features are described here, and full details are included in the File S1.

**Figure 1:**
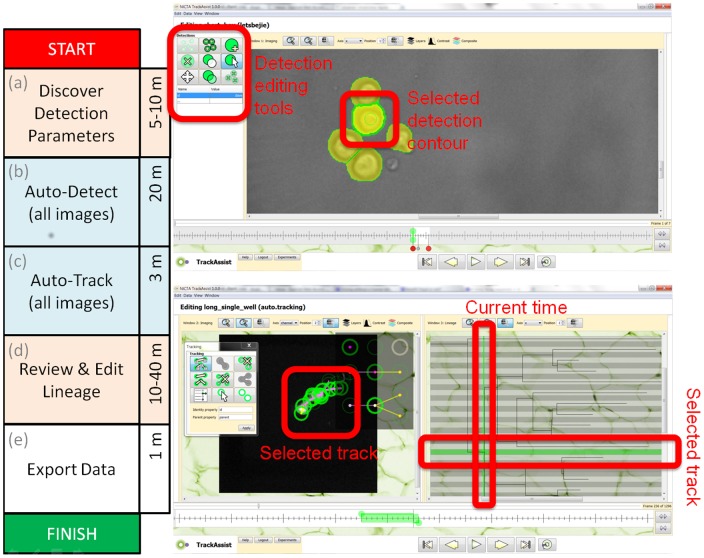
Workflow for the processing of a population of cells in a single microwell, resulting in a pedigree analysis. Data produced includes contours, tracks and lineage for all cells in all images. The timeline on the left shows tasks in order and the average time required for the results in this paper. The blue areas are automated tasks, with no user interaction required. Pink areas are manual operations. On the right side of the figure are two screenshots of the user interface during the two time-consuming manual tasks. Top: Automatically segmented detections as shown during the process of evaluating segmentation parameters, overlaid on the bright field channel (NB: Fluorescence channel is used for segmentation, bright field allows user to validate). Below: track editing while simultaneously viewing the lineage context of each cell-detection. The selected track is highlighted in both imaging and lineage views. Both views update simultaneously in response to edits. The lineage tree allows users to find and fix suspicious division, death and track start events very quickly.

Users of our software reported that a multi-view interface was most crucial feature, whereby the same solution could simultaneously be viewed in lineage and imaging modes (see figure 1). Edits in either view immediately updated both. Due to the heritable qualities of lymphocyte division and death, the lineage view was particularly useful for identifying incorrect track fork and track end events, the most common types of error. The imaging view was always needed to verify the correct sequence of events. Note that the software allows different time points and image types to be displayed simultaneously (for example, “display the fluorescence channel 5 images in advance of the transmission image and at double zoom”).

The design of the user interface was a combination of video-player and image-editor software conventions. We provided typical Video-Cassette-Recorder controls for playback of image sequences, with extra buttons to control speed and repeat short loops. The interface gives the user the ability to annotate any pixel in any image. The annotations appear as text-boxes in the images (but are non-destructive to the underlying image), and as balloons on the timeline, allowing them to be easily found; in practice we found users made heavy use of annotations.

In the imaging views, the most critical features were: the ability to apply a nonlinear enhancement to contrast; to superimpose detections and tracks over a window of time (past and future); and to composite channels (e.g. using a separate colour for each different image shown).

Generating lineage has two precursor steps. First, detection of cells in all images. Second, association of cell detections as tracks. The key user interactions for detection were: An auto-detect tool that finds all cells in a range of images, with preview of results; a delete-detections tool that can remove “all selected detections” or, “all detections within a range of images”; a detection-selection tool; and some brush tools for adding or removing pixels from detection contours. The vast majority of detection was done automatically, with the other tools used to fix segmentation errors where necessary. Automatic cell segmentation was achieved using some basic image pre-processing followed by the Watershed algorithm [41].

Tracking was also almost entirely automated, with manual tools for fixing tracking errors. We provided tools for joining, forking, and splitting tracks. The automated tracking method is a combination of the Extended-Hungarian approach from Kachuouie et al [42] and the concept of “object existence” from LJIPDA [43].

## Results

Three types of data are presented. First, we report the effort and time required to produce this data. Second, we present evidence that our results are valid by reproducing population statistics from [5]. Third, we attempt to use our data to generate some novel results. To this end, we trained a classifier to predict cell fate and present some new observations of lymphocyte size, speed and lineage made possible by the large quantity of cells tracked.

### 2.1 Labour efficiency

The data produced in this paper was generated by three users of the TrackAssist software over a period of two weeks. We selected all microwells with initial populations of one or two cells and then tracked all progeny until all cells died. For each microwell, we recorded the time required to complete each stage of processing. The workflow adopted is described in section 4.3 and figure 1. Almost all effort was consumed in two stages: cell segmentation and track correction. (Lineage is an implicit result of tracking in our model). The factors influencing time required are the number of images and the number of cells. We captured images of each microwell at intervals of 5 minutes for a total period of 108 hours, resulting in 1296 images per microwell.

The typical population of a microwell was 4–12 cells and the maximum microwell population was 53 cells. In total 44 founder cells were tracked resulting in 350 dead progeny, with a total of 675 distinct cells tracked. To clarify, if a microwell had an initial population of one cell, that divided once, there would be a total of 2 dead progeny and 3 cells tracked. All cells were tracked throughout their lifetimes with no tracking losses (automated tracking failures were all manually corrected).

We found that using the semi-automated methods implemented in the TrackAssist software it was possible to perform all segmentation and tracking activities more quickly than existing manual methods used in [5]. The methods reported in this paper also scale better than earlier methods (see figure 1), making it practical to study microwells with more than one founder cell and final well populations of up to 32 cells. An approximate guide to the effect of software automation was that labour for a complex microwell with population more than 20 cells was reduced from one week to three hours (see table 1). A more detailed analysis of the labour efficiency results can be found in the section 3 of File S1.

**Table 1:** Comparison of labour required (in minutes) to track all cells in a microwell and extract lineage, using either ad-hoc manual methods (such as spreadsheets, Matlab etc.) or the TrackAssist software.

Microwell max cells	1 to 4	5 to 8	9 to 16	17 to 32
Ad-Hoc manual methods	15	120	600	1440
TrackAssist (active time)	6	23	38	147
TrackAssist (total time)	27	45	61	180

Note that manual time does not include time spent generating visual aids such as custom video files to support manual activities. The manual methods scale poorly as the number of cells increases. When using the TrackAssist software, a distinction is made between “active” time (when the user must interact with the software) and total time (including time the user is simply waiting for the computer). The user can perform other tasks while waiting. The total number of images was 1296 per channel (transmission and fluorescence). TrackAssist time values given are mean averages from 4 users over 675 cells and 42 microwells. Ad-hoc times are consensus opinion of authors of [5] with reference to lab notes.

### 2.2 Validation: Reproduction of existing results

**Figure 2:**
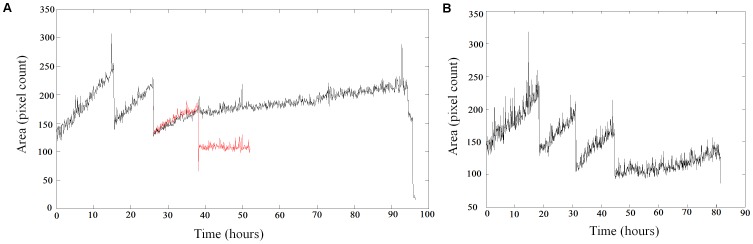
This figure is a reproduction of figure 2 in Hawkins et al [5]. However, we have selected two abnormal profiles (A) and (B). Each panel shows cell area over time, across several generations, for a single lineage. High frequency variation in cell area is due to changes in illumination, morphology and measurement noise. Cell growth causes a gradual increase in area. Each time a division occurs, cell area drops suddenly and one of the daughters is used to continue the series. (A) shows the area profile of a lineage that is believed to have undergone a failed division event at approximately 38 hours. Two lineages are plotted, in red and black. They share the same ancestors for 2 generations, and have a similar growth profile in the third generation until our postulated “failed division”. Thereafter, one lineage (red) shows a further division giving a daughter that does not grow or divide. The other lineage (black) fails to divide at the same time and continues to grow, albeit more slowly. (B) shows a lineage in which fluorescence increased dramatically prior to death (from about 63 hours) without a corresponding increase in transmission image cell area. Automatic segmentation falsely dilated the cell area. One possible explanation is that GFP production continues (possibly with less regulation) in some cells even after the division machinery ceases to operate.

Although our approach to *in vitro* microwell lymphocyte tracking is faster for non-trivial microwell populations, it is not useful unless is it also accurate. Since we have produced tracks for a large number of cells it is not feasible to check all these tracks manually. Instead, we have reproduced the population and lineage statistics from Hawkins et al [5]. Figures 2, S1, S2, S3 (in File S1) show reproduction of Figures 2,1,[Fig pone-0083251-g003] 4 and 5 in Hawkins et al respectively, using our data. Although the experiments were conducted in slightly different conditions, we found good agreement with the original paper. Notably, we found similar parameters for fitted distributions and similar correlations between related cells' division times. These correlations also agree with other existing studies e.g. [24]. Correlations are a sensitive and measurable proxy of tracking accuracy. If our software produced more tracking errors than the manual approach, we would expect that correlations between sibling division times would decrease. In particular, erroneous track-swaps would reduce sibling correlations.

**Figure 3 pone-0083251-g003:**
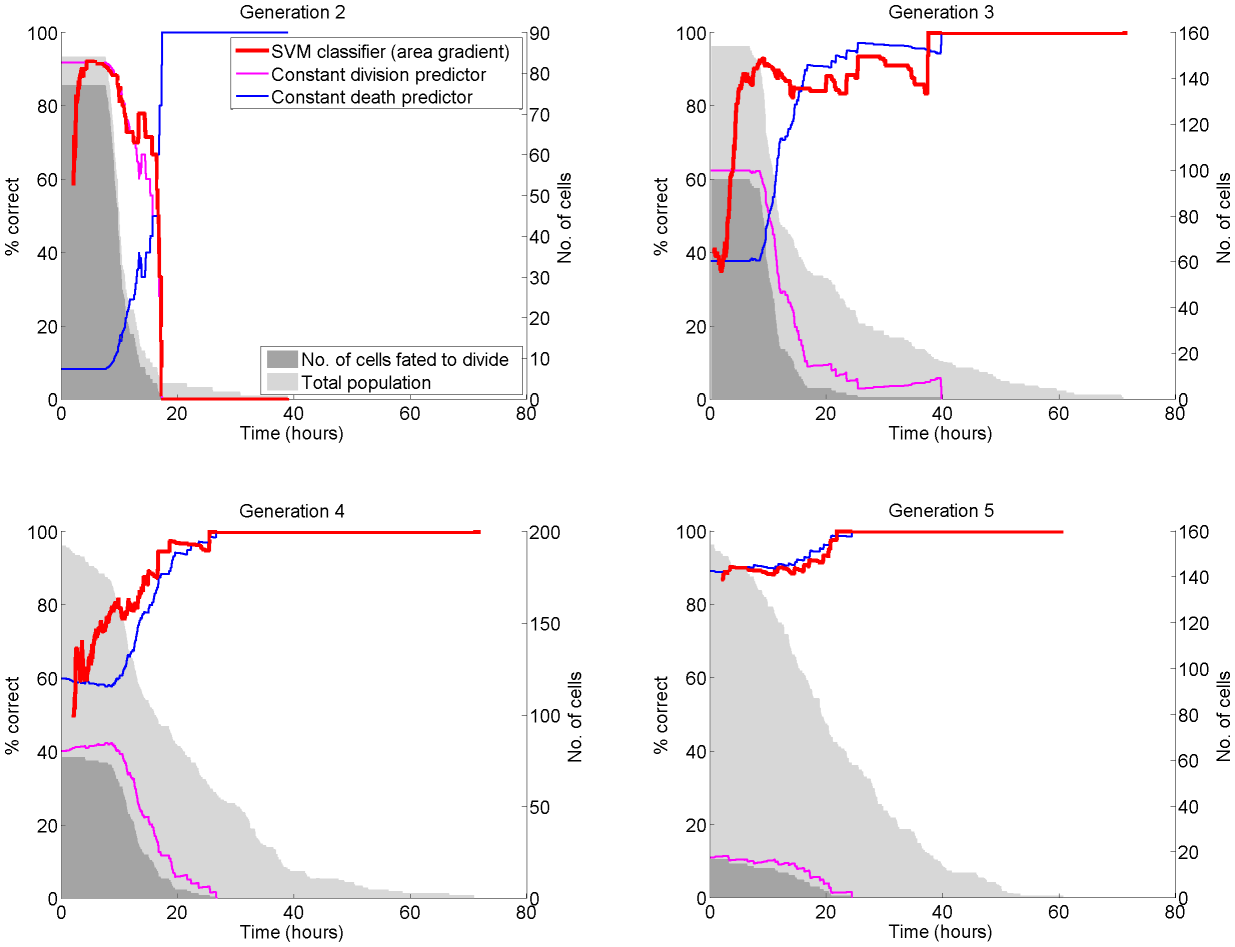
Predicting the fate of tracked cells by measuring growth. We used a simple SVM classifier to predict the fates of lymphocytes given the gradient of their observed area over time. Gradients were calculated from a rolling window of area measurements. In some generations we were able to predict cell fate with more than 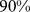 accuracy shortly prior to the majority of divisions and before almost all deaths. The fate of every cell was independently predicted (series shown in red) at the time of capture of every image given data about its observed area produced by tracking. Since in many generations or after most division events the majority of cells have the same fate, we also show the prediction accuracy possible by predicting all cells die (blue series) or divide (pink series). We expect the SVM predictor to perform better than either constant-fate prediction. To be useful, the classifier must correctly predict fates before they occur. A period of observation is required to measure growth accurately, but these results show that for the majority of cells enough measurements are available prior to most division events. In many generations (including those not shown) there was insufficient training data, or the samples were highly skewed to a specific fate. These factors reduced classifier accuracy; with more data, it is likely that better classification could be achieved. We have included less successful results such as generation 5 to illustrate the effects of highly skewed training data. Since we used only 33% of data for training, the classifier in generation 5 had only 6 training samples for the dividing fate.

**Figure 4:**
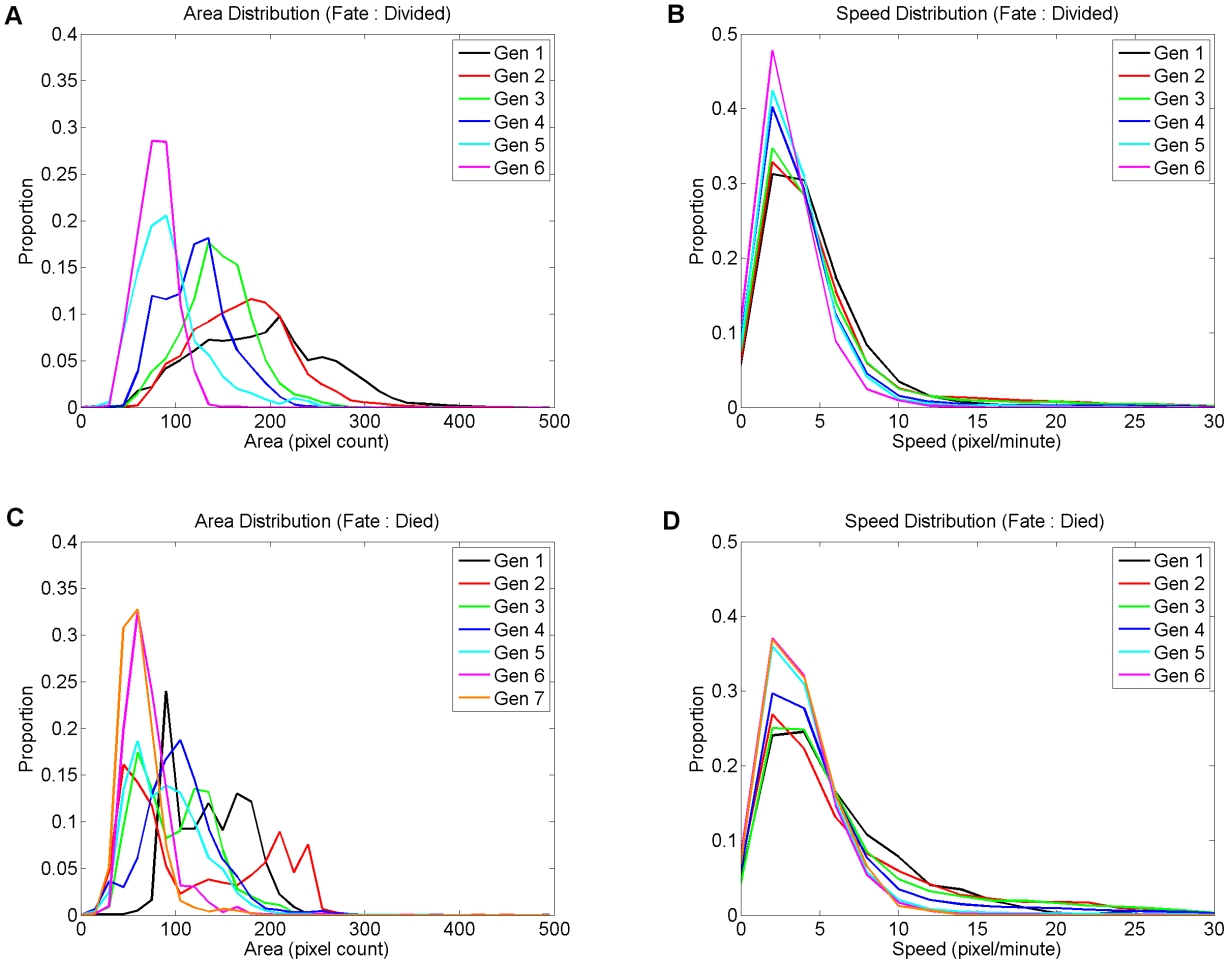
Histograms of cell area, and speed, for each fate and generation. Cell areas and speeds are computed as mean values over the lifetime of each cell. Since the total number of cells in each category varies, values shown are proportions of category total. Figures A and C show histograms of area, and figures B and D show histograms of speed (motility). These results suggest that cells fated to divide tend to move more slowly with each successive generation, and that cells fated to die tend to move more quickly than cells fated to divide. As expected, cells fated to divide tend to be smaller with each generation of division, but the relationship between area and fate is less clear for cells fated to die. In some generations dying cells' size follows an unexpected bimodal distribution. It is possible that the bimodal results are a consequence of systematic segmentation bias caused by intensely fluorescing dying cells (see section 2.4).

**Figure 5:**
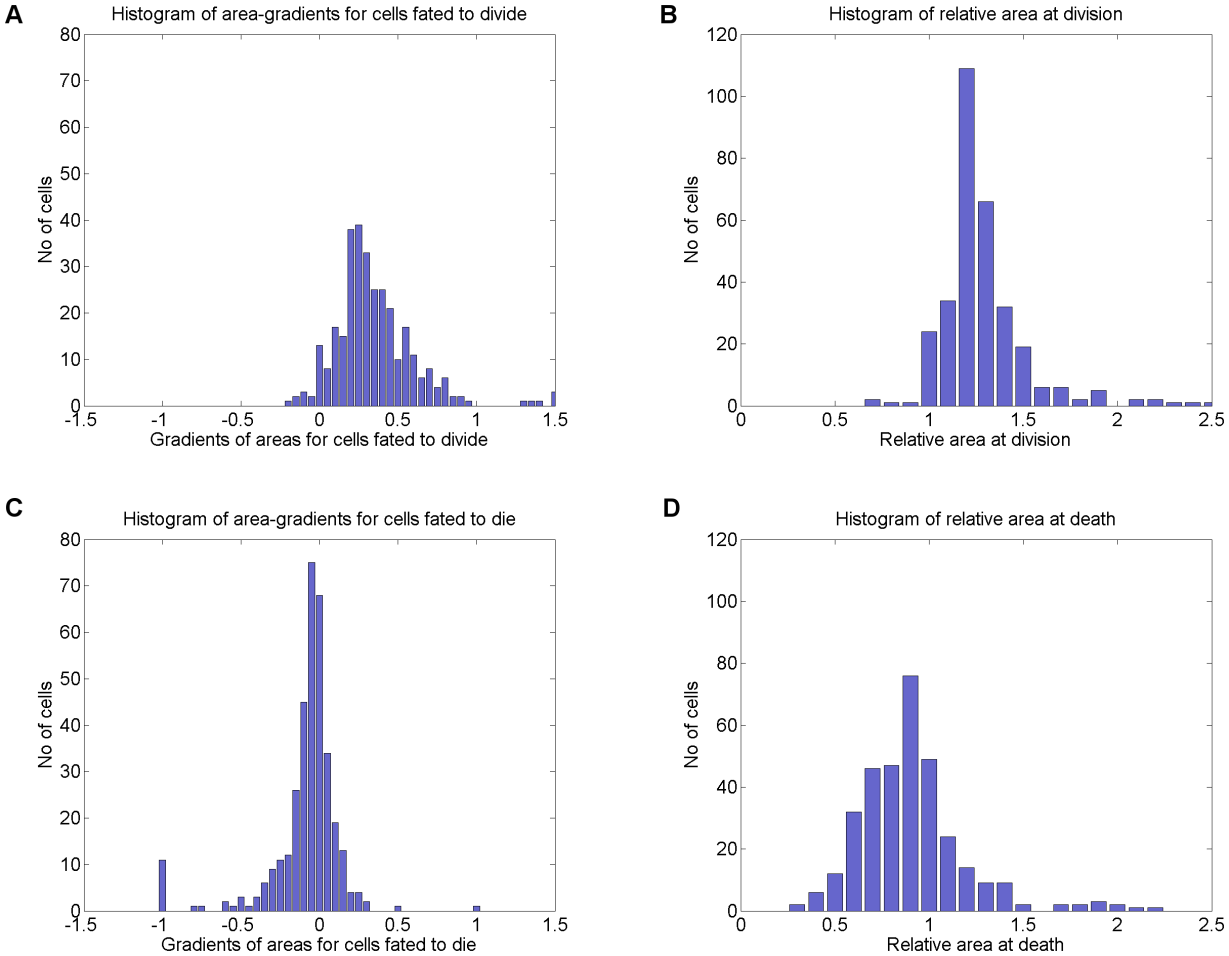
Histograms of lymphocyte area-gradients and relative areas between birth and death or division. Area-gradients were computed as the difference between cell areas in two windows, each of duration 3 hours. The windows were fixed at cell birth and cell death or division. A median filter of size 13 was used to remove outliers. Relative area was computed similarly, using median-filtered windows of duration 3 hours at either end of the cell's lifetime. Relative area was computed as the ratio of the two windows' median values i.e. a relative area of 2 means that area doubled. “Area gradient” quantifies the rate of growth and “relative area” gives a measure of total growth achieved. Previous pedigree studies of lymphocytes have not tracked a large quantity of cells making distributions less informative. Although, as expected, dividing cells tend to grow and dying cells tend to shrink, approximately 8% of cells fated to die evidence some growth (defined as a relative area at death in excess of the mean plus one standard deviation). Measurement noise explains the majority of these; others showed some growth followed by a period of zero growth. 3.75% of cells were measured as growing substantially (relative area in excess of mean plus two S.D.), producing a second mode centred on a relative area of 1.9 (see D). Verification in the bright field revealed that these cells did not actually grow but instead fluoresced more intensely, resulting in a systematic segmentation bias.

Other types of error should also be evident in these population statistics. If our software over-segmented the cells, we would observe a greater number of divisions and deaths due to transient false tracks. Under-segmentation would also produce a greater number of deaths and divisions when cells are temporarily combined as a single track, before appearing to split again (a false division). We did find some discrepancies between our data and Hawkins et al. These are discussed in the section 4 of File S1.

### 2.3 Utility: New lymphocyte spatial data

Our automated approach has produced a much greater quantity of *in vitro* B-lymphocyte time-lapse tracking spatial data than previously available. In particular, this has provided some more subtle observations of lymphocyte motility and area while controlling for lineage, fate and generation. This quantity of data would not have been practical without labour-efficient, interactive automation. We present these observations as examples of the types of cell population modelling made possible by the techniques in this paper.

#### 2.3.1 Lymphocyte fate prediction

The authors are not aware of any successful use of phenotype to predict the fate of individual cells. However, this is an interesting area of research since there are a broad range of applications, including high content screening [2]–[4] where the phenotypic classifiers used for fate prediction might serve as an alternate readout of drug effect. The importance of fate prediction in the case of neural progenitor cells was discussed in Cohen et al [44]. The authors developed a segmentation and tracking algorithm based on [45]. They call their method AIPT and predict applications in a variety of fields. One particularly interesting use is to isolate cells with particular qualities, which could assist genetic analysis of the genes responsible for those qualities. Timm Schroeder has written a review of the potential value of cell fate prediction, not only death or division but including ultimate cell type and function [1]. Schroeder describes the use of time-lapse live cell imaging for this purpose.

We found that the fate of most individual lymphocytes can be predicted using tracking. We trained and tested a Support Vector Machine (SVM) classifier to predict cell fate in each generation of our lymphocyte data. Using 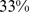 of the data for training and 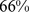 for testing, we were able to predict between 80–90% of individual cell fates correctly prior to the actual fate of 90% of cells (depending on generation, see figure 3). In other words, it was possible to predict most fates before they happened. The predictions were generated from a small moving window of cell area measurements prior to the majority of cell divisions (i.e. fate was predicted for almost all cells before fate was observed). Our results show that in all generations more than 90 percent of lymphocyte fates can be predicted in advance using cell area-gradient, and training on a large dataset. Since we did not provide generation as an input to the classifier, our results apply to experiments where generation is not available. Using generation (as a prior), higher accuracy could be expected.

The method of computing area-gradient 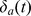 for a single cell at time 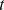 is given in equation 1. The gradient is taken as the difference in automatically segmented area between the current time 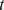 and a prior time 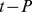. To smooth the measured areas, cell area measurements 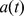 are summed over a window of radius 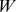:
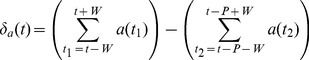
(1)


Our method of fate *prediction* was entirely automatic, but was trained on data that had been manually corrected using TrackAssist. The quantity and quality of tracking data resulting from our semi-automated approach is necessary for training cell behaviour prediction. Using a realtime tracking system with fully automatic cell fate prediction could allow *in vitro* segregation or other intervention prior to cell division, death or expression of a phenotype characteristic. The methods described in this paper are fast and accurate enough to allow automatic realtime intervention.

Some caution should be applied to interpretation of these results for the following reasons. First, the data was heavily skewed in some generations towards a particular fate. Accuracies of approximately 85%, 60%, 60% and 90% are possible in generations 2,3,4 and 5 simply based on the prior probability of one fate. However, our classifier performance was able to exceed this prior probability in all generations, substantially in generations 3 and 4 where both fates occurred with high frequency. The second reason for caution is that we tested our classifier accuracy at each time point, producing a series of classification accuracy values (figure 3). In novel experiments under different conditions, the optimum time to apply the classifier might not be known. Earlier classification results in lower accuracy because cells haven't yet grown; later classification loses value because many cells' fates have already occurred. We have described here the optimum classification performance prior to the determination of more than 90% of cells' fates. In all generations we were able to achieve classification accuracy in excess of 90%, but in some generations only after more than 10% of fates had occurred.

Improvement of fate prediction using cell area was hampered by small and heavily skewed training data in some generations. In generations 2 and 5 the majority of cells have a common fate. The proportion of cells that will die in a given generation could be provided to the classifier as a prior and would likely increase prediction accuracy. However, in this paper we wished to show that we can predict more accurately than is possible using only the prior probability of a given fate in each generation. Therefore, we show the accuracy possible by constantly predicting either fate as a reference, and are able to predict much more accurately than this in generations 3 and 4 (figure 3). In generations 2 and 5 it was not possible to significantly improve on constant-fate classification, because very few cells in training and testing data had the alternative fate. More measurements could improve prediction: in [44], cell centroid, area, eccentricity, major axis length, and orientation were considered. Although in our experiments lymphocyte proliferation is sufficiently delayed for us to observe every division, in other protocols cell generation data may not be available.

#### 2.3.2 Lymphocyte area, generation, and fate

 1. *Lymphocyte area is smaller and less variable with successive generations, for cells fated to divide*.

Figure 4 (panels A and C) shows histograms of cell area by generation. Separately plotting cells of differing fates reveals some interesting observations. For cells fated to divide, as expected, modal cell size decreases with each generation. Variance in cell size also decreases with successive generations.

2. *Bimodal distributions of cell area for Lymphocytes fated to die*

For cells fated to die, the data are less clear (figure 4 panel C). There is no strong ordering of the means or variances by generation. In generations 1, 2 and 3, histograms of dying cells' areas do not show unimodal distributions. Instead, bimodal histograms are observed. A substantial part of the dying cell population (up to 40%) is in the smaller mode in some generations. By contrast, for cells fated to divide, the area histograms are unimodal. The distributions of cell area for cells fated to die are dissimilar to cells fated to divide, in all generations except 4 and 5. The bimodal distributions cannot be explained by cell segmentation bias due to varying fluorescence (see section 2.4), because these artefacts were only found in a tiny fraction of the total population. Further, we identified the segmentation bias by looking at cells with growth before death. The histograms of dying cell area-gradients for these generations are unimodal, not bimodal. It is not simply that some dying cells grow - or are measured as growing - prior to death. A number of large cells from early generations are fated to die despite their size. These cells are abnormally large, compared to the mean size of cells from either fate, in generation 2. These mixed results suggest there may be multiple causes of cell death, whose relative prevalence varies by generation.

#### 2.3.3 Lymphocyte motility, generation, and fate

We found some interesting and consistent variations in lymphocyte motility when grouped by generation and fate. While recognizing that *in vivo* motility will likely be different due to surfaces that aid movement, fluid medium and intercellular influences, the internal characteristics of lymphocytes are the most likely cause of phenomena observed. More importantly, *in vitro* motility could still be useful as a proxy or predictor for *in vivo* behaviour or phenotype. In the following results, motility is assumed to be the distance between cell centroids between successive images. Centroids are computed as the centre-of-mass of the automatically segmented, connected pixels (further details is in the section 4.1). The impact of cell shape changes on measured motility has not been considered.

1. *Lymphocytes fated to divide tend to move more slowly in later generations*

Figure 4 (panels B and D) shows histograms of cell speed by generation. Although the distributions are not grossly different by fate or generation, there are some interesting observations. Cells fated to divide tend to move more slowly in later generations. There is a gradual shift in mass from the high-speed tail of the distribution to the slower mode. Each successive generation shows an incremental shift towards slower motion. Again these observations could be used to assist classifiers or predictors of cell fate or phenotype and were not previously reported due to the difficulty involved in generating enough data. Even a weak signal can be incorporated in a multivariate prediction algorithm.

2. *Lymphocytes fated to die tend to move faster than those fated to divide*

In all generations cells fated to die tend to move faster than cells fated to divide. This was unexpected as greater lymphocyte motility is associated with an increased immune response [14].

### 2.4 No evidence of genuine cell growth in final generation

Figure 5 shows histograms of cell area-gradient and relative area at division and death. The area-gradient represents the rate of growth; relative area gives scale of net change. Measurements at the start and end of cells' lifecycles are computed over a window of 36 frames (approx. 3 hours) to reduce noise. Panels A and C show that, as expected, cells fated to divide tend to grow, and are larger at division (typically 1.25× their original area according to panel B). Cells fated to die do not grow, with most shrinking. However, a small number of cells fated to die appear to show substantial growth prior to death. This was not reported by Hawkins et al [5], [46] who noted that in the clones where cell size was measured over time no growth occurred after a cell had “decided” to die. On this basis they reached the conclusion that cell fate was decided early in the cell cycle.

With any distribution of values, some values greater than the mean would be expected even if only due to measurement noise. The relatively high proportion of cells showing small positive gradients or slightly larger relative areas may be simply measurement noise. However, 3.75% of cells fated to die were measured as having substantial growth that is unlikely to be measurement noise. For the purpose of this discussion substantial growth is defined as a relative cell area at death of 1.4 or greater, this threshold being 2 standard deviations above the mean relative area of 0.9. In panel D it can be seen that a small mode of cells were measured as growing to nearly twice their original size before death.

Our results were generated by segmenting the cells in the fluorescence channel. We investigated the 3.75% outliers by visually comparing their segmented contours in with their appearance in both fluorescence and transmission image channels. Some dying cells did grow, but we believe these cells were really “failed divisions” because they showed a pattern of rapid growth, followed by a period of slower growth (see figure 2). In these cases, the change from rapid to slow growth also coincided with division of the sibling cell.

**Figure 6:**
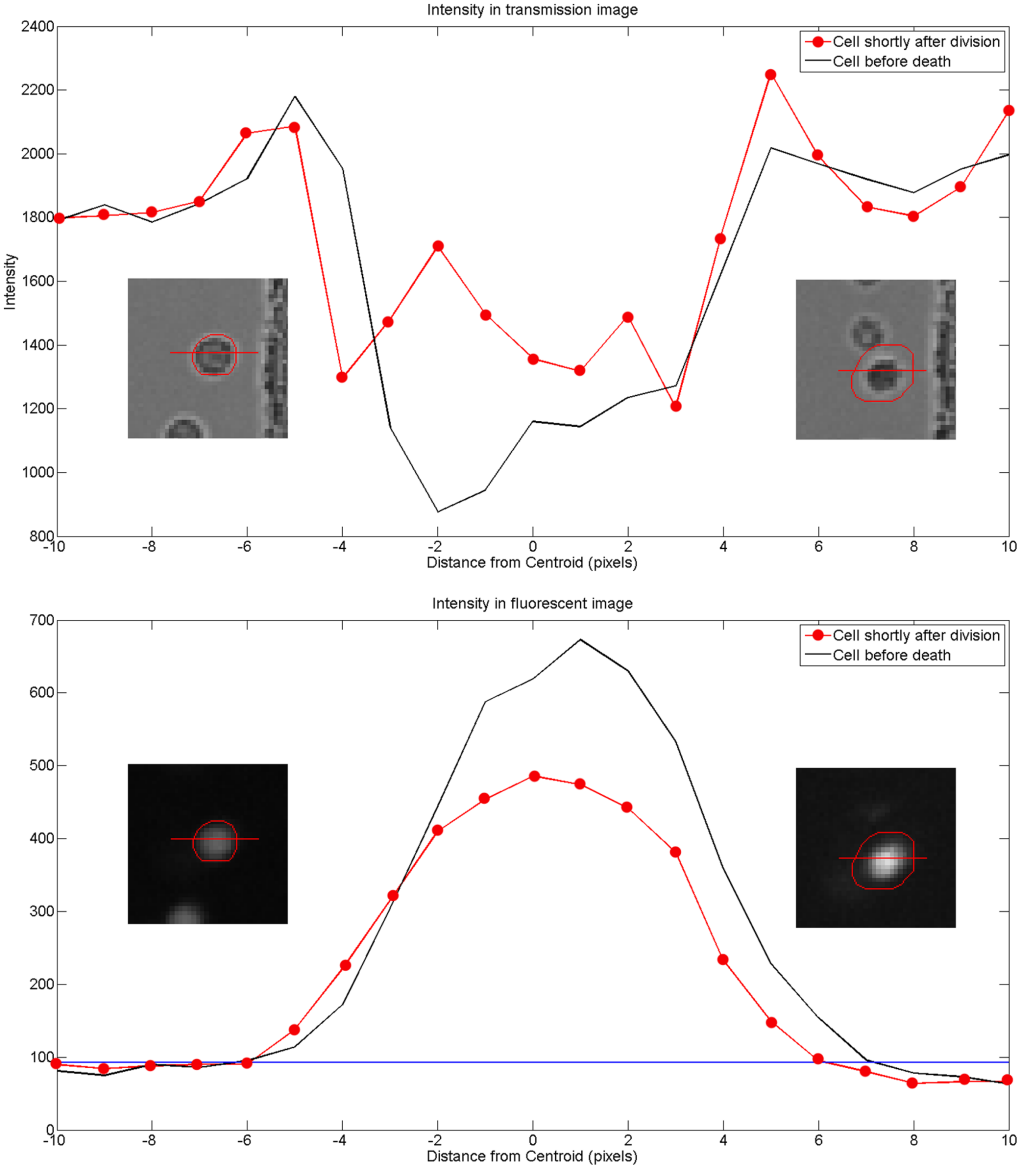
Systematic cell segmentation errors caused by increased fluorescence intensity in some dying cells. This data is generated from images of one cell measured having substantial growth but which eventually died rather than divide. Plotting image intensity along cell cross-sections allows more precise localization of cell boundaries. While in the transmission images growth cannot be substantiated, it is interesting that the fluorescence image shows significant increase in intensity and fluorescing area, that appears to extend beyond the boundary of the cell. The red contours were generated by TrackAssist based on difference to normalized background intensity. The dilation of the segmented area appears to be a genuine feature of the cell, but no longer representative of cell area.

Other dying and growing cells had segmented contours that were not corroborated by the transmission image (see figure 6). The growth in these cells was therefore a systematic error of our segmentation algorithm. This is a good example of the ways that automated methods can produce misleading statistics. We found that some dying cells start to fluoresce more strongly than normal. In addition to any blurring caused by the point-spread function of the microscope optics, the intensity of the fluorescence is partly a function of the thickness of the slice of the cell in the focal plane. In consequence, fluorescence is greatest nearer the centre of the cell and least near the edges of the cell. If the cytoplasm is uniformly brighter, a greater fraction of the cell area passes the threshold test causing the contour of the segmented cell to dilate.

After a thorough manual review of all cells showing significant growth we could not find any cell that convincingly showed any growth in the final generation, therefore our results strongly support an early determination of cell fate within the cell cycle. The evidence is particularly strong due to the quantity of cells that we tracked over several generations. Unlike genuine growth leading to cell division, the onset of increased fluorescence in dying cells is late in the life of the cell. This meant it did not significantly impact on our ability to predict fate.

### 2.5 Sibling growth correlations

This data is provided because it has not previously been shown (for example, in previous publications).

1. *Rate of growth in siblings both fated to divide strongly correlated*

**Figure 7:**
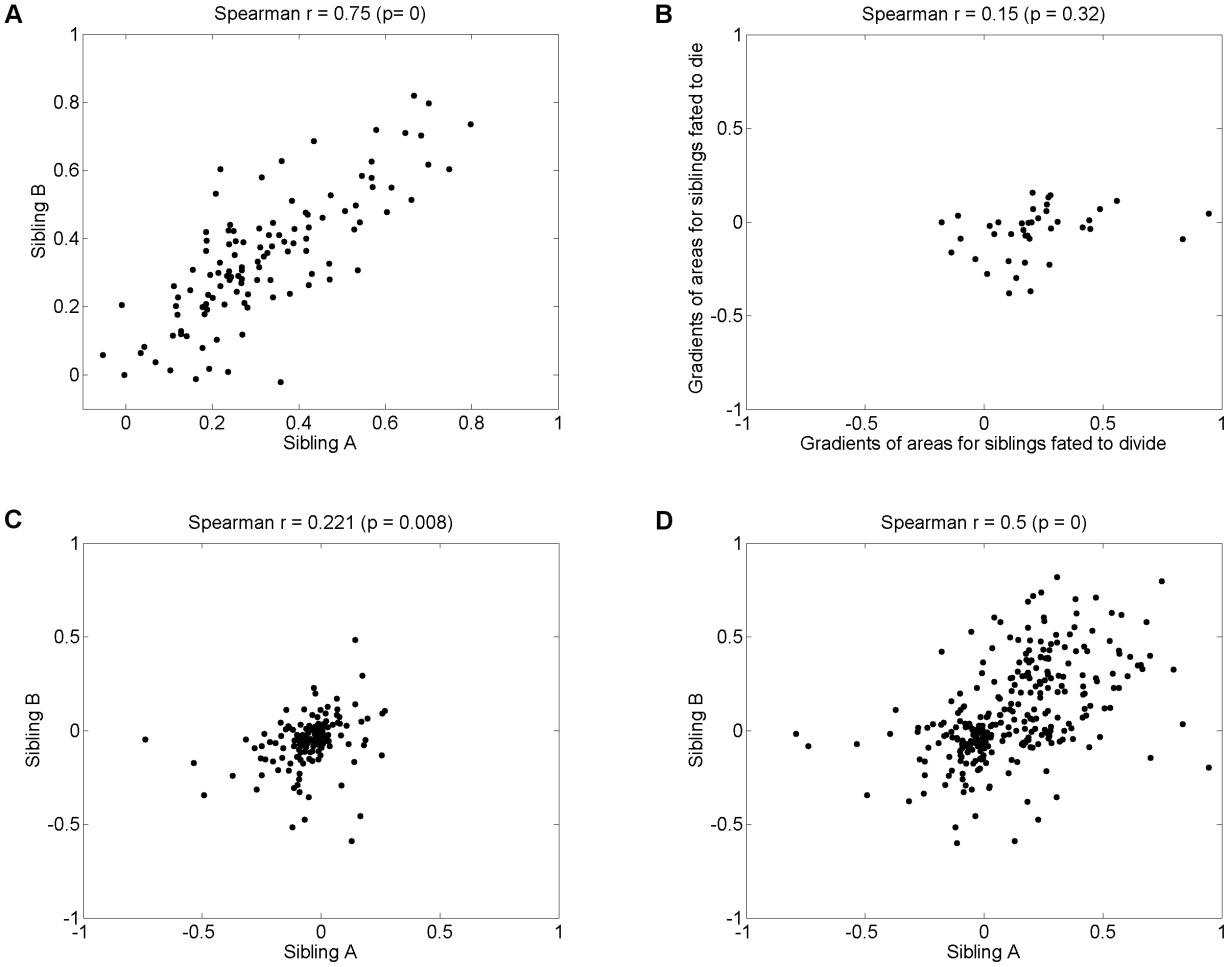
Correlations between area-gradients of sibling cells. The area gradient is computed by applying a median filter (size 12 measurements) over each track's size measurements, to reduce noise. A line was then fitted to the resultant points and the gradient of the line is reported. This measures the rate of growth rather than overall size attained. Regardless of generation, there are strong positive correlations between siblings when both siblings divide (A). When one sibling dies and another divides (B), the rates of growth are only weakly correlated. Since the gradient is computed throughout the life of the cell, this suggests that fate determines cell growth early in the life of the cell reducing growth of the dying cell compared to its sibling. In the case of both siblings dying (C), both siblings rates of growth are clustered around zero with a slight positive correlation, although as discussed in section 2.4 any apparent growth in the final generation may be an artefact of increased fluorescence intensity and automatic segmentation. Panel (D) shows correlations for all fate combinations, an intermediate correlation as expected because the division correlation is diluted.

Figure 7 shows correlations between the area-gradients of siblings. The area-gradient is the rate of change in cell area over time. Ignoring fate, there is a weak positive correlation between growth rates of siblings (panel D). However, when different fate combinations are shown separately a more informative picture emerges. Panel A shows a strong positive correlation between siblings who both divide.

2. *Rate of growth in siblings not correlated for other combinations of fates*

In the case that one or both siblings do not divide, their rates of growth are not correlated (see figure 7 panels B (one dies, one divides) and C (both die)).

## Discussion

The software used for these experiments has been released under the GPL2 open source licence and is available at: github.com/NICTA/TrackAssist.

Installation instructions can be found at: github.com/NICTA/TrackAssist/wiki/Install/wiki/Install.

Raw data from these experiments (excluding images) are available at: github.com/NICTA/TrackAssist/tree/downloads/tree/downloads.

The data includes the contours and other properties of all detections and tracks.

The most striking result from our data was that we were able to predict individual lymphocyte fates with useful accuracy using only observations prior to the division or death of almost all cells. We also observed that *in vitro*, lymphocytes tend to slow down in later generations and that lymphocytes fated to die tend to move more rapidly than lymphocytes fated to divide.

These results would not be possible without the quantity of tracking and lineage data that can be produced using semi-automated methods. Although we predicted fate, it is likely that other phenotype characteristics could be predicted with the same technique - first, semi-automated segmentation and tracking of a large number of cells to construct a database of cell behaviour, and second, training a classifier on this data for automatic prediction of cell fate or phenotype in subsequent experiments. A system of this type would have significant applications in high content screening, or to execute experiments that require intervention based on phenotype, such as segregation of some cells for treatment or analysis.

For the first time we have published quantitative information about the effort, time and methods required to produce this type of data from time-lapse *in vitro* live cell imaging experiments. Although many researchers have extensive experience of these issues, it is rare for them to be documented sufficiently to allow other researchers to accurately estimate the impact of potential problems. We intensively tracked a greater volume of lymphocytes than previously reported, which was only possible due to efficiency improvements.

Compared to the strategies adopted previously, considerable time was saved. As found by Rapoport et al [38] and Scherf et al [39], the quantity and “quality” of our results was improved by the interactive and semi-automated approach we took: We have a richer set of data that includes contours, tracks and lineage for every cell. By reproducing published population statistics, we have provided evidence that our results are not only of greater volume and quality, but are also very accurate. As already discussed, this data can be mined for features that identify new phenotypes or to train classifiers or predictors of cell behaviour. We see no reason why this approach cannot be generalized to other experiments.

The automation methods, graphical tools, and workflow we describe all contributed to the practicality of the data analysis we have reported. We argue that to generate this type of data efficiently and accurately, all these factors must be considered and appropriate tools should incorporate these features. Currently, Imaris [30] is the only commercial tool offering good graphical track interaction tools, but even that product does not allow simultaneous editing in both lineage and imaging perspectives.

We identified a systematic bias affecting the size of cells fated to die. The bias was due to a minority of cells fluorescing more intensely while dying. This is a good example of the type of error that can be expected when using automation, and shows why automatic results should be manually verified using effective interaction tools. The existence of a relationship between fluorescence *intensity* and measured cell *area* may not always be obvious, but will likely occur in many experiments if pixel intensity thresholds are used to segment cells.

### 3.1 Further work

Although we have focused on cell area, growth and motility there are a number of other characteristics that could be used to inform tracking and achieve higher automation accuracy. For example, lymphocytes can be treated to fluoresce in different wavelengths (colours) indicating the onset of particular internal events. While cell area and the intensity of fluorescence are not constants, they change relatively slowly and so these measurements can inform association of cells between frames.

In our software there is limited interaction between the automation and the user. Currently, we allow the user to configure the automation but not otherwise interact with it. A better strategy might be to allow users to place constraints on the model and repeatedly apply the automation to unconstrained parts. For example, the user could “lock” partial or complete tracks regardless of whether they were produced manually or by automation. The automation would then improve the remaining tracks given this knowledge. Allowing users to explicitly limit automation objectives is also promising, such as “blacklisting” of repeated automation errors. Suitable problem-specific representations and user interactions would be the focus of research in this topic, because *implementation* of tracking constraints has been thoroughly studied. Tracking could even become a background task triggered automatically after every user intervention. This could lead to an effect similar to the spell-check function in many word-processing packages, where issues are immediately highlighted for user review.

Segmentation could also be improved by more intelligent tools that exploit users' intelligence in an efficient way. Graphical tools can be designed to facilitate “knowledge transfer” from the user to the computer, by providing hints that software learns to associate or interpret in terms of image data. For example, existing imaging-based cell segmentation tools ask the user to click inside the cell and then try to infer the boundaries from image data. This approach has worked well in a number of image interpretation domains, such as segmenting roads or counting trees in aerial imaging (Zhou et al [47], [48]). Their assistive semi-automated methods were found to reduce user time required by 40–76%.

The authors are optimistic that some of the assistive, interactive software methods given above might help to boost researcher productivity, potentially leading to richer results and more complex insights into cell biology and population dynamics. In many cases, all necessary technical solutions are available and lack only suitable interface development to enable application to specific problem domains.

## Materials and Methods

The data reported in this paper was produced by culturing lymphocytes in microwells. Microwells are physical partitions that prevent small groups of cells from mixing. Each microwell is effectively a separate tracking problem. For easy segmentation, lymphocytes were sourced from transgenic mice whose cells continuously express GFP (a Green Fluorescing Protein). Two images (transmission and fluorescence channels) were captured every 5 minutes over a period of 108 hours resulting in 2 sequences of 1296 images of each microwell. Full details of the raw data that was produced can be found in the section 1 in File S1.

Processing of this data was undertaken using a piece of software developed expressly for this task, named TrackAssist. Instructions for downloading, building and running this software can be found at: github.com/NICTA/TrackAssist/wiki/Install.

In this software, cells were automatically segmented using the GFP channel, due to the high intensity of cells against an otherwise dark background. Automatic segmentation was followed by automatic tracking (and implicitly, lineage recovery). Finally, user interaction with the software was used to correct tracks (and if necessary, segmentation) until an accurate result was obtained. A thorough description of the segmentation, tracking and user interaction features of the software can be found in File S1.

### 4.1 Automation: Cell segmentation

**Figure 8:**
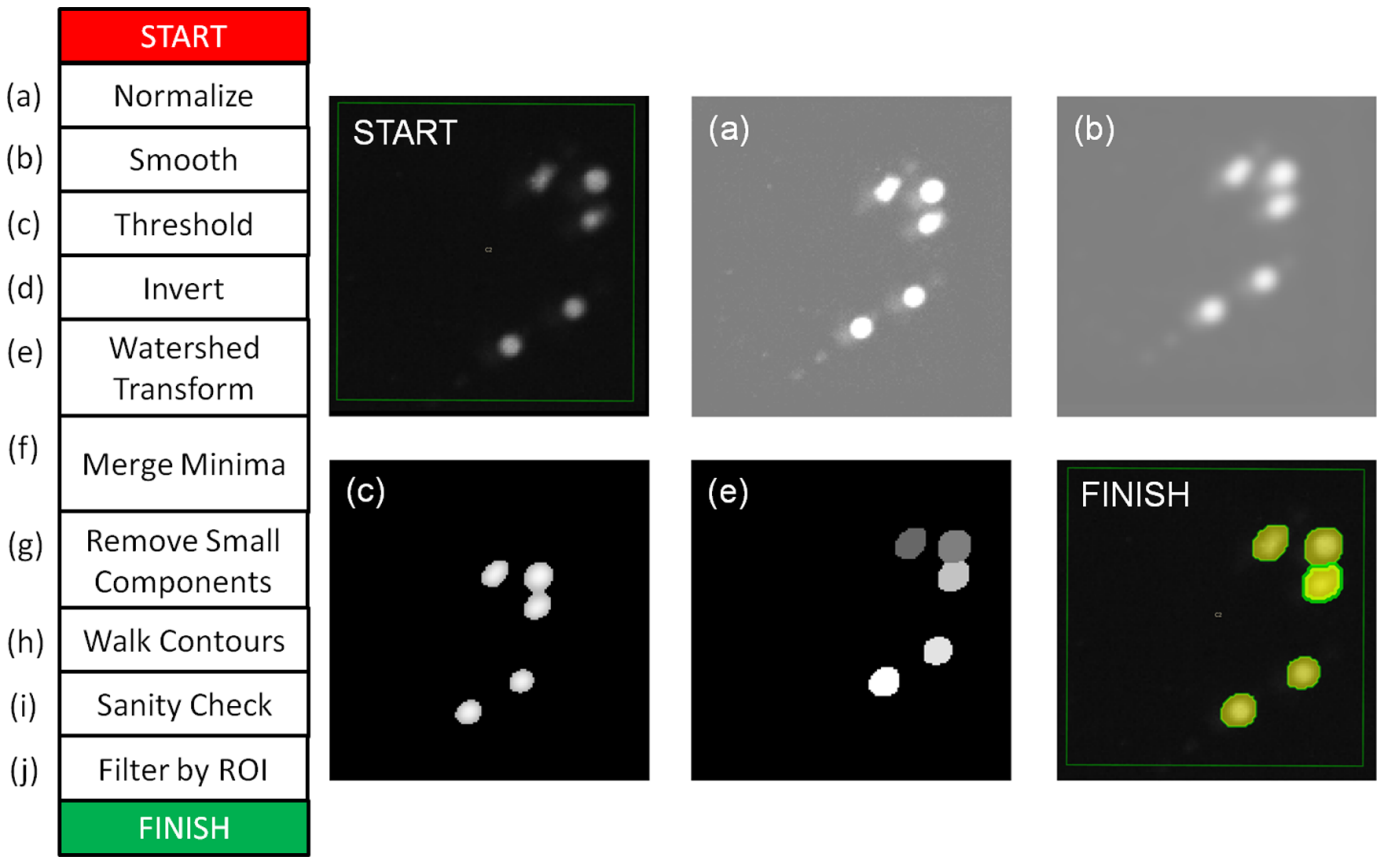
Process for automatic detection and segmentation of GFP cells that constantly fluoresce. This figure is provided to give the reader a more detailed understanding of the steps involved. The image labelled “START” shows 5 cells in a single microwell, with the user-defined Region Of Interest (ROI) shown as a green box. The images are normalized (a), smoothed (b) and thresholded (c) at a fixed intensity level. Intensity levels above the threshold are preserved, allowing the Watershed algorithm and connected-component analysis to label pixels associated with each individual cell (labels shown in image (e)). The Watershed step is necessary to segment cells that are touching. The remaining steps (f–g) try to control over or under-segmentation. The sanity check (i) exists to detect bad images due to acquisition faults. Bad (white noise) images produce an implausible number of detections and are then skipped. The final segmented contours are shown in (“FINISH”), with one cell selected.

Cells were automatically segmented using the GFP channel, due to the high intensity of cells against an otherwise dark background. Figure 8 shows the process used to segment constantly fluorescing cells. Each image was processed independently. Each input was a 16-bit greyscale image 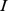. (a) Images were independently normalized to give pixels a mean intensity of 0 and a standard deviation of 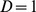, resulting in image 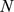. (b) Images were smoothed by 2-d convolution with a Gaussian kernel with sigma 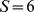. The kernel sigma was determined by cell resolution and size in pixels.

The range of intensity values were then scaled to the interval 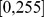, giving image 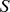. (c) A clipped intensity image 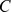 was produced from 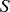 by thresholding with a user-specified parameter 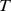; pixels with intensity less than 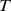 were assigned a value of 0 (denoting background). Ideally, cells will have an intensity value other than zero, whereas non-cell areas will have a value of zero. Note that due to the method of normalization, a single intensity threshold can be used throughout an image sequence assuming that the intensity histograms of the images do not change significantly.

The remaining steps are designed to separate lymphocytes that are touching each other (i.e. “de-clumping”). The method is based on the assumption that fluorescence intensity is greatest in the centre of each lymphocyte and decreases towards the perimeter. First, (d) the clipped image 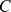 is inverted, such that the brightest parts of cells have the lowest values. This image is then passed as input to the Watershed transform (e) [41], which associates each foreground pixel with the nearest minima (i.e. nearest cell-centre). The association is performed by producing a labelled image (see figure 8) in which each pixel's value is the label of the connected component (i.e. cell) it belongs to. In the event that minima (cell centres) are much closer than minimum cell size, they cannot be separate cells. Therefore, we merge connected components where the minima are less than 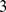 pixels apart (f). We also remove components with a total area less than another threshold 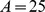 pixels (g). Finally, the contours of the remaining components are walked using the Moore Neighbourhood algorithm [49] (h).

The final part of image processing deals with some camera malfunctions and and user preferences. Occasionally, the camera will capture an image without illumination, resulting in white noise and no signal. This causes image processing to produce a very large number of small cell detections. We skip processing these images by comparing the number of detections in the previous image to the number of detections in the current image. If there are more than 10× the number of detections in the second image, it is assumed that a camera fault has occurred and all detections are rejected (i). Finally, we allow the user to specify a Region of Interest (ROI), typically one microwell, and discard cells detected outside this area.

### 4.2 Automation: Cell tracking

**Figure 9:**
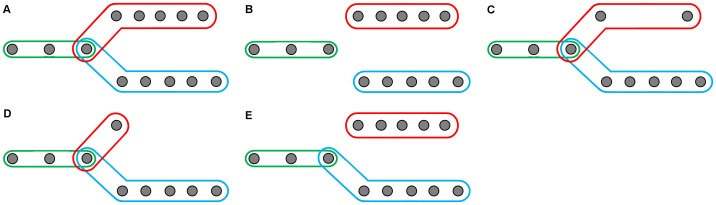
Modelling of tracks and lineage. We represent lineage implicitly by allowing a detection to be included in more than one track (A). Orphans are tracks without parents (B). Sparse tracks (C) have few detections over a long period of time. Short tracks (D) have few detections in total. False splits (E) occur during automated and manual track editing, and can be converted into a single longer track.

Cells are detected in each frame independently. Detections of the same cell over time are associated forming tracks, therefore a track represents a sequence of cell detections over time. For cell tracking, we wish to use tracks to infer lineage. We chose to represent lineage implicitly, by allowing detections to be associated with more than one track (see figure 9 A). This means that tracks do not split or merge. Instead, some tracks share detections to model lineage. For example, the final detection of a mother cell track will also be the first detection in each of the two daughter cell tracks.

To construct lineages of cell tracks, we first associate detections into track fragments without allowing splits or merges (figure 9 B). Track creation is allowed at any time point. We subsequently prune tracks to remove any that are very short, or very sparse (see figure 9 C,D). We also remove false-splits, where exactly two tracks share a detection (figure 9 E).

**Figure 10:**
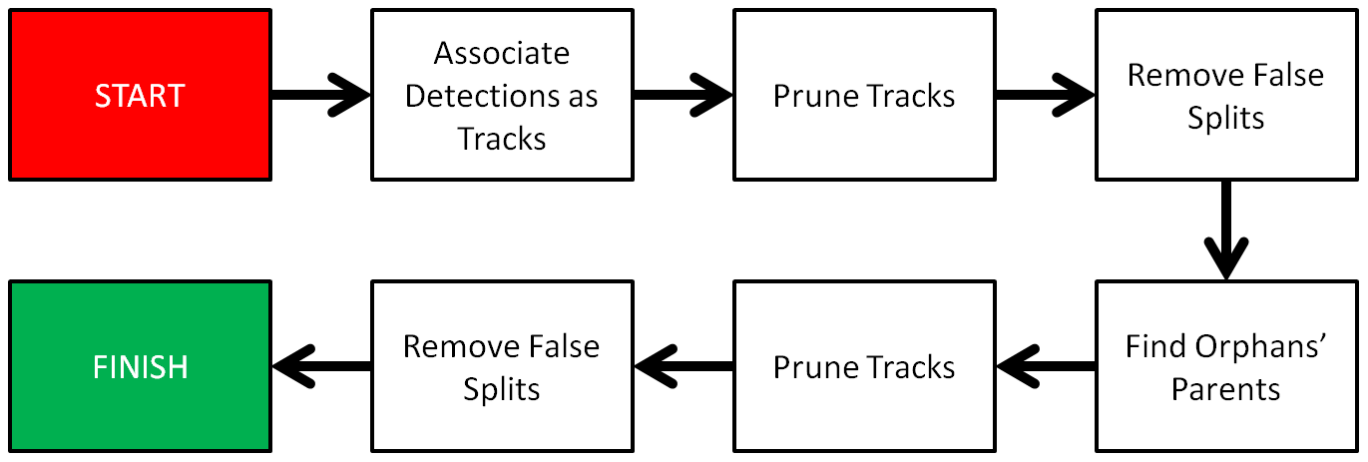
Flowchart illustrating process for automatically tracking cells, with lineage constraints.

After these steps, it is necessary to assemble track fragments into lineages. We expect that orphan tracks are very unlikely to be observed after the first few frames, because microwell walls prevent cells from moving into view. Therefore, we try to find sequential links between track-fragments and candidate cell divisions to explain the set of tracks that are observed. These merge operations may also generate false splits, short tracks and sparse tracks that need to be pruned. The end result is the lineage. An overview of the complete tracking process is shown in figure 10.

#### 4.2.1 Detection and track association

A family of algorithms based on Bayes' theorem have been widely and successfully applied to tracking problems [50]. These algorithms iteratively update a probability density function of objects' state (position, velocity, area etc.) using measurements from each successive image. In our case, objects are cells. Mathematically, this posterior density is defined as 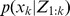, where 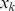 is the state at time 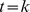 and 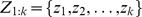. 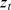 is the detection collected at time and 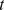 is the collection of all detections up to time 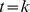. The posterior density can be obtained from predicted density and a measurement likelihood function:
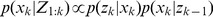
(2)


The two terms on the right hand side in (2) are likelihood and predicted density respectively. The predicted density of the state depends on the dynamic nature of the object (how the object moves in each time interval). On the other hand, the likelihood term captures the fidelity of the detector (how accurately has the detector detected the object). Together, these two terms contribute to the posterior state density.

More practically, the detector may miss the target and, in addition, may detect false objects. The simple likehood, described above, does not capture these types of sensor properties. In PDA, [51] these properties were introduced by the probability of detection 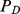 (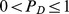) and by modeling false detection density respectively. These parameters affect the likelihood and thereby the state density (from 2).

The tracking of cells is essentially a multiple-object tracking problem. Hence, additional hypotheses are required to accommodate the presence of multiple objects and the reality that a detection can originate from any of the objects in the vicinity (or none in case of missed detection). This problem was elegantly resolved in the Joint PDA (JPDA) algorithm [52].

JPDA weights the likelihood of a detection associated to an object based on the likelihoods of the same detection to other objects as well as to the event that the detection might just be originating from none of the objects. Finally, JPDA provides an weighted mixture of all hypotheses as the final posterior density of the object state.

However, there are problems in using the JPDA algorithm for tracking cells, [42]. Some of these problems include the way cells stick together, and in the case of time-lapse microscopy, the large sample interval. Therefore, in [42], the authors used the likelihood of JPDA and introduced an optimal assignment algorithm to associate detections to objects (instead of weighted mixture of all detections) in order to calculate posterior state density. The proposed algorithm, Extended-Hungarian-JPDA, was shown to have improved performance.

However, the use of JPDA lacks in one important aspect. The algorithm has no innate capacity to distinguish between a true track (a track following a true object with defined dynamic model) and a false track (a track that might have been initialized incorrectly from a false detection). Therefore, it depends on heuristics such as “N missed detections over M time samples” to decide whether to retain or terminate a track. This is not a systematic solution, i.e. for example it does not differentiate between a long established track and a short spurious track.

The Joint Integrated PDA (JIPDA) ([53]) and its computationally efficient version, Linear LJIPDA ([43]) introduce the concept of “object existence” as a random event and proposed a systematic way to calculate the probability of that event. The existence of an object 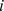 at the time 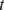 is given by 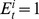 while its complementary event is given by 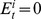 (the object does not exist). JIPDA or LJIPDA calculates the likelihood as in standard JPDA discussed above except that the likelihood values will be additionally weighted by the probability of existence of each object (the long established objects will get priority when a detection is to be assigned to several objects in the vicinity). LJIPDA then iteratively updates the probability of existence. This forms the basis of automated “track management” namely, if 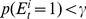, the track gets terminated (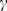 is a suitably chosen value between 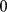 and 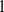).

In our software, we combined the Extended-Hungarian approach from [42] and the concept of “object existence” from LJIPDA ([43]). This LJIPDA-Hungarian tracking algorithm overcomes the common problems in cell-tracking applications (low sampling rate, clustered cells etc.) and also provides flexible track management capabilities through the existence probabilities of each track, which allow us to model the population constraints of microwell *in vitro* cell cultures.

We assumed a Brownian model for cell motion. The tracking module receives the centroid of each detection (collected from the segmentation process) and tries to associate each detection to one of the existing tracks using the LJIPDA-Hungarian algorithm. It then updates the probability of existence for each track and decides whether to retain or terminate the track. We use 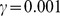 as the threshold to terminate a track. Therefore, the software resulted in high quality tracks and terminated spurious tracks very early.

#### 4.2.2 Lineage constraints

The tracking algorithm produces a list of track segments where each track is a collection of one-to-one correspondences between cells in adjacent frames. This formulation of the problem allows us to leverage the target tracking literature to obtain a robust tracking algorithm [50]. Cell divisions and the construction of the lineage trees are inferred from the track fragments. Due to the physical confinement of cells within microwells all cells must be either a founder cell or a descendant of a founder. Therefore the start of a new track segmented in any frame other than the first implies a division. In a typical cell division event, the mother and one daughter will already be associated as a track fragment, with the other daughter being represented as a new track. The orphaned daughter can be re-associated with her mother by finding the most suitable cell detection in the image immediately prior to orphan track initiation.

A distance function is needed to compare potential mothers. An obvious choice is pixel distance, but it is not robust to image scale changes and does not model uncertainty in the locations of the detected cells. We use the Mahalanobis distance between the position of the first detection of the new track and the position predicted by the motion model for each existing track:
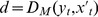
(3)where 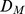 is the Mahalanobis distance, 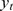 is the location of the first detection of the orphan track, 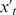 is a Gaussian distribution representing the motion prior at time step 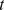 (i.e. the posterior at time 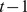 propagated through a Brownian motion model.) The track with the smallest 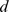 that satisfies 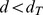 is chosen as the mother track (i.e. under extreme circumstances orphans are allowed).

Note that more accurate association of mother and daughter cells could probably be achieved by consideration of cell size, and conservation of total cell volume (the daughters' volumes should be approximately equal to the mother's volume prior to division). However, allowing the cell size to influence lineage, which in turn is used to produce statistics on cell size, risks biasing the results. While nothing in the experiment can be modelled as completely independent statistical processes, it is clear that cell position is relatively independent of cell size.

### 4.3 Workflow

We will describe the workflow we used to generate our results in the times reported. The unit of work is considered to be building a complete description of all lymphocytes in one microwell. This includes detecting the contours of all cells in the microwell in every frame, and association between all these detections generating a complete lineage.

Figure 1 outlines the tasks involved in processing one microwell. The first stage is automatic segmentation. Although the process is automatic, on a new dataset it can take a few minutes to test a variety of parameters until good segmentation is achieved. The software supports parameter evaluation by allowing tests on one or more images before application to the entire sequence. Additionally, the results of each stage of image processing can be previewed for the current image. The steps involved in automatic segmentation are described in figure 8.

We found that a 50-frame test window was suitable to determine good parameters for automatic segmentation, because illumination, microwell population and cell sizes change during imaging and affect thresholds. In practice, this task takes between 5 and 10 minutes, though it could probably be automated [54]. Once good parameters are found, they can be applied to the entire sequence. The resultant detections were reviewed by scanning quickly through the sequence to look for an unusually high or low number of detections, or detections that looked too large. Rarely, a second round of parameter tuning would be required. No manual correction of detections was performed; instead, bad detections were simply excised from tracks. Individual detections not part of any track had no impact on our results as they were ignored.

**Figure 11:**
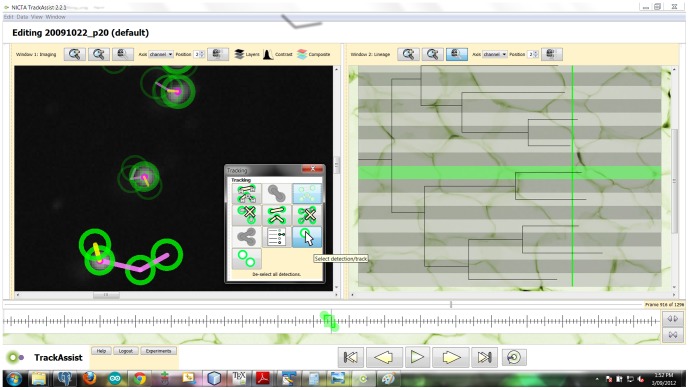
Graphical user interface of the TrackAssist software. This figure shows one imaging window on the left (many can be shown at once) and one lineage window (right). The lineage window has a vertical green line indicating the currently viewed time point and a thick horizontal green bar indicating a single selected track. The selected track is visible on in the imaging window. Tracks are currently shown as a “window” of 4 detections, 1 future (yellow), and 3 past (purple). The current detection has a dot in the centre. Green circles indicate the size of each detection. The past and future time window is controlled by moving the green circles in the time control (bottom). The tracking tools are also visible. In row-major order from top left, these are: Annotated tracking; associate tracks & detections; de-select all tracks & detections; delete selected track; automatic tracking; delete all tracks; fork tracks; tracking legend; select tracks & detections; separate selected tracks & detections.

The second stage is automatic tracking. The default parameters were used in every case. The third and final stage is manual track review & editing. After automatic tracking, the lineage window was used to determine where to edit tracks (see figure 11). Rather than review every track from end to end, we only looked at divisions and terminations. Every division and termination was reviewed by replaying those events over a window of approximately 5–10 images with tracking overlays. Where errors were found, they were typically mis-associations due to division or death events - most commonly, assigning the children resulting from a division to the wrong parent. (A better software would consider cell size when assigning division products to parents.) Errors were fixed by separating tracks into parts at the moment of mis-association, then joining the track halves correctly. Once all division and death events have been reviewed, the microwell is complete.

### 4.4 Interaction

This section will describe the tools we provided for users to interact with the detection, track and lineage models necessary for the type of spatial and temporal analyses given in this paper. To avoid the common issue of having a near-perfect automatic solution and no tools to fix the last few errors, we decided that all data produced by the software must have full options for manual editing and review, so that any error can be fixed if the user decides it is worth the effort. We provide these descriptions to describe the minimum set of features we needed to allow efficient manual review and correction of cell tracks & lineage in time-lapse microscopy. A thorough description of the tools is also necessary to understand the workflow we adopted, which is detailed below.

#### 4.4.1 Detection editing tools

**Figure 12:**
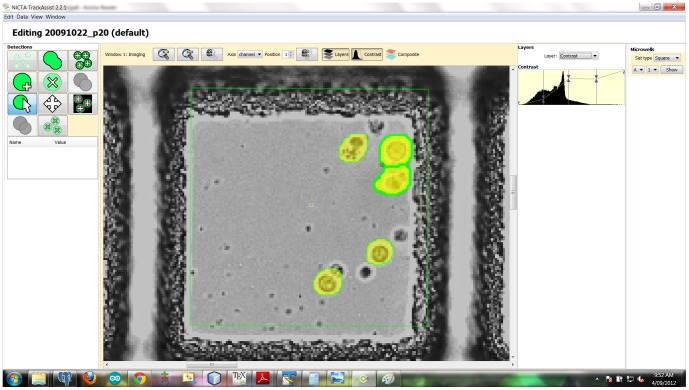
Graphical user interface of the TrackAssist software. The bright field is shown here, with histogram based manual contrast enhancement for viewing purposes. Five segmented cells are shown, two of which are selected. Other objects in the well are debris. The detection editing tools are shown in a panel on the left. In row-major order from top-left, these are: De-select all tracks and detections; merge selected detections; automatically create detections (by background modelling and motion detection); create detection; delete selected detections; cut brush from selected detection contour; select detections; move detection; automatically create detections (based on thresholded constant fluorescence); add brush area to selected detection contour; delete all detections in range of images.

Using tracking terminology, a detection is one observation of a cell in a specific image. TrackAssist displays detections as a translucent area bounded by an 8-way connected contour. This is natural for small objects where rasterization effects are significant and the accuracy of segmentation can be measured to a resolution of one pixel. By colouring the contours in an unnatural way (figure 12), they are clearly visible regardless of the zoom level of the image.

We provide a detection *selection* tool, that toggles the selection status of detections when they are clicked. Therefore, an arbitrary set of detections can be simultaneously selected. A *de-select-all* tool is provided for convenience. New detections can be created with a special tool, based on a circular brush (since most cells are approximately circular). The contours of detections can be improved using *brush-add* and *brush erase* tools, which add or subtract a variably sized circular area from a selected cell. We originally intended to add other brush shapes but found this unnecessary in practice.

A *merge* tool allows a set of selected detections to be combined into a single contour. This is useful for improving over-segmented results. A move tool allows detections to be moved intact although we found this less useful. Often, while experimenting with automatic detection of cells, bad parameters cause a large number of detections that must be deleted. A *delete-all* tool allows the removal of all detections in a range of images (by default, the current image). Another option is to *delete all selected* detections.

We provide two tools for automatically detecting cells. One, not covered in this paper, used cell motion to segment lymphocytes from background microwell appearance. The other automatic detector assumes that lymphocytes constantly fluoresce, and is described in figure 8. Note that even given 95% accurate automatic cell detection, the automated method is only a small part of the software functionality required to efficiently segment cells. A small set of interactive tools is more complex to implement.

#### 4.4.2 Track editing tools

We provided an *annotated tracking tool* as an alternative to automatic tracking. This tool is useful for quickly recovering lineage information without reconstructing tracks in detail. The tool is similar to the Metamorph tracking tool [22] used in Hawkins et al [5] and tools described in [23] and [21]. The approach involves manually tagging cells at the start of imaging, and at all division and death events. We require that each cell is tagged at least at start and end (and as often between as desired). Each tag of a cell must give the cell a unique ID. At least once, each cell must have a tag giving the cell's parent's ID. If the mother tag is absent, the cell is assumed to have no known parent. Using these tags, complete lineage and sparse tracking models can be computed directly.

Since tracks are the association of detections over time, it is very useful to be able to see past and future detections overlaid on the current image and current detections (see figure 11). We provide a time-window control to allow the range of past and future detections displayed to be adjusted independently. Detections shown are selectable regardless of whether they are in the current image or not. This allows a set of detections to be selected and associated as a track with the *associate* tool. Similarly, a *separate* tool allows a set of selected detections to be dis-associated. Existing tracks can be associated with additional detections as long as there are no overlaps in time.

Tracks are represented by circles centred at each detection, with straight lines between the centres (see figure 11). Track starts, terminations and divisions all have differently coloured dots on the relevant detections. The number of detections in a track that are visible is determined by the time window control. Tracks can be selected by clicking inside the circles of any detections belonging to the track.

We also provide a lineage view (figure 11). This perspective shows all tracks but not orphan detections. Tracks' selection status can be toggled by double-clicking on the relevant rows in the lineage view. Note that track selection applies to both lineage and image views, allowing the lineage context of tracks to be associated with their appearance. Since there are many ways to manipulate both track and detection selection status, a de-select all tracks and detections tool is provided for convenience.

Tracks can be deleted with a dedicated tool. All selected, or all tracks can be deleted.

We represent lineage implicitly by having tracks' detections overlap (explained in figure 9). However, the software normally forbids detections being associated with multiple tracks. Therefore it is necessary to provide a special tool to join tracks to define their lineage. The *fork* tool requires the user to select 3 tracks. One track must terminate before the others begin and is designated the mother track. The last detection of the mother track is added to the other two tracks. To undo a fork, the *separate tracks* tool can be used to remove the linking detection from all 3 tracks, before re-associating it with the mother.

Finally, an automatic tracking tool is provided. Although some parameters are adjustable, the tool is essentially automatic. The methods used were described in section 4.2.

## Supporting Information

File S1 **Supporting text, figures and tables.** The file contains data acquisition methods, detail of different visualization features of the software, labour efficiency result, tables and figures to support various conclusions and observations.
(PDF)
