# Supplementary material for: Labour-Efficient In Vitro Lymphocyte Population Tracking and Fate Prediction Using Automation and Manual Review
Source: PLoS One. 2014 Jan 3;9(1):e83251. doi: 10.1371/journal.pone.0083251 (PMC3880260; doi:10.1371/journal.pone.0083251)
Supplement: File S1 — Supporting text, figures and tables. The file contains data acquisition methods, detail of different visualization features of the software, labour efficiency result, tables and figures to support various conclusions and observations. (PDF) [file pone.0083251.s001.pdf]

Supporting Information

1 Data Acquisition

In our experiments we used grids of microwells [1] to physically partition lymphocytes into non-mixing groups and prevent them from moving out of camera view. Partitioning simplifies the tracking problem, because the number of potential associations is greatly reduced. Partitioning also increases long-term tracking success, because individual lymphocytes and their progeny are likely to be visible for the duration of the experiment. Each microwell is effectively a separate tracking problem.

For easy segmentation, lymphocytes were sourced from transgenic mice whose cells continuously express GFP (a Green Fluorescing Protein). The lymphocytes were put into culture and stimulated with CpG 26 hours prior to image capture. The lymphocytes were cultivated in 8-well Ibidi chamber slides. 125 micron PDMS microwells were placed into each chamber. Two images (transmission and fluorescence channels) were captured every 5 minutes over a period of 108 hours resulting in 2 sequences of 1296 images of each microwell. A Zeiss Axiovert 200m microscope with Axiovision software was used to automatically capture images. Temperature was controlled at 37 deg and 5% CO2 was maintained.

To capture the fluorescence images we used a Zeiss HBO mercury arc lamp with a 6% reduction filter and Zeiss Filter Set 13, 1s exposures. The transmission (bright field) images were captured with 50ms exposures taken through Filter Set 13 with a GFP filter cube. Then objective lens was a Zeiss FLUAR 10x/0.5 & #8734;/0.17 1.9mm 440135-000 1. Images had size 1388 x 1040 pixels with a resolution of $0.65\mu m$ per pixel.

2 Visualization

The data enhancement and display control features of our software tool are described below. We include these to provide insight into the type of graphical tools and visual aids that are useful in various image interpretation, tracking and time-lapse microscopy studies. We expect that this list of features can assist researchers developing similar tools, by providing ideas and guidance on useful graphical conventions and interaction tools.

2.1 Compatibility

The software we developed, named TrackAssist, reads images directly from typical automated microscopes. These images are usually 16 bit TIFF format, which can be read using the ImageJ API [2]. The utility of any microscopy software is maximized if the original images can be immediately viewed without conversion or re-encoding using third-party tools.

2.2 Annotations

The software includes the ability to add text annotations to any image. This allows users to associate their thoughts, interpretations and other unanticipated comments with a particular feature in an image (figure S4 shows an example). Annotations are pinned to a particular pixel in the images and are non-destructive (i.e. the image underneath is not affected). The annotations are moveable, editable and can be deleted after they are created. When images are zoomed, the annotations remain a normal font size. Any text can be added to the annotations. Annotations can be exported, allowing users to keep their comments associated with their data. Annotations appear in the time-line control (figure S4) allowing them to be easily located later. Although simple, we believe that this feature has great value in any image-interpretation tool.

2.3 Pan, Zoom and Contrast

Due to variations in experimental conditions, some form of contrast enhancement is also essential. It is also useful to be able to zoom and pan images without smoothing to maximize the precision of observations (see figure 12 in the main article illustrating these features). These features are very similar to the capabilities of image editing software such as Adobe Photoshop or Gnu Image Manipulation Program (GIMP) [3]. Often, features such as these are neglected in research-oriented image processing or tracking software. A good ability to arbitrarily zoom, pan and enhance is essential for interpreting image content. Some subtleties are important. For example, zooming by a constant scale factor (e.g. 0.5x, 2x, 3x etc.) is far less useful than a nonlinear scaling. We used a power function to scale image pixel size x using base $z = 1.1$ and integer levels y starting from 1:

$$x = z^y \tag{1}$$

This formulation provides a good compromise between rapid zooming and the ability to fit a variety of content to the screen at the highest possible resolution. For contrast enhancement, we provide a user controlled 5-piece linear mapping of the input intensity range with an intensity histogram to help the user decide how to optimize the mapping for most informative display (figure 12 in the main article).

2.4 Navigation of time

In time-lapse microscopy it is also necessary to navigate through sequences of images in time order. Tracking requires association of objects between image frames and to manually verify a set of associations it's often necessary to move back and forth repeatedly observing the same events. We have provided several user-interface components dedicated to tracking interpretation of time-lapse images. First, we provide the ability to scroll backward and forwards in time by individual frames, using the mouse wheel. Next, we provide two controls for selecting specific time points: A conventional "slider" control provides gross time selection within the entire time sequence, and a custom time control with tick-marks provides fine control. When sequences have length greater than 100 images, both systems are helpful.

2.5 Playback

We also allow various playback tools. The user can define a window of time in which playback is looped over the same events again and again. Playback speed can be adjusted, and playback can be in either direction. To further improve the visualization of associations between detections over time, we overlay the image at a particular time point with track and detection information from a window of time in future and past (discussed below).

2.6 Colour Compositing

It is common for time-lapse microscopy experiments to capture multiple images of the same viewpoint at the same time using a variety of filters and illumination sources (for example, variously coloured fluorescence versus bright field). Studies often use fluorescence to indicate the state of cells or the presence of activity within them. It is therefore very useful to be able to change the view between the different simultaneous images without losing pan, zoom and time position. Another frequently used visualization is channel "compositing", in which multiple greyscale images are overlaid, using separate colour channels for each (see figure S5 for an example).

3 Labour Efficiency Results

The data produced in this paper was generated by three users of the TrackAssist software over a period of two weeks. We selected all microwells with initial populations of one or two cells and then tracked all

**Microwell max cells**	1 to 4	5 to 16	17 to 32
**Processing Stage:**	Minutes	Minutes	Minutes
Active segmentation	5	12	15
Passive segmentation	20	20	20
Passive tracking	1	3	9
Active track correction	1	26	132
Total **active time**	6	38	147
Total time	27	61	176

Table S1. Cell tracking time requirements, per microwell. All times in minutes.

progeny until all cells died. For each microwell, we recorded the time required to complete each stage of processing. The workflow adopted is described in section 4.3 and figure 1 in the main article. Almost all effort was consumed in two stages: cell segmentation and track correction. (Lineage is an implicit result of tracking in our model). The factors influencing time required are the number of images and the number of cells. We captured images of each microwell at intervals of 5 minutes for a total period of 108 hours, resulting in 1296 images per microwell.

The minimum population of a microwell was 1 cell and the maximum microwell population was 53 cells. In total 44 founder cells were tracked resulting in 350 dead progeny, with a total of 675 distinct cells tracked. To clarify, if a microwell had an initial population of one cell, that divided once, there would be a total of 2 dead progeny and 3 cells tracked. All cells were tracked throughout their lifetimes with no tracking losses (automated tracking failures were all manually corrected).

The times required for tasks have been divided into “active” and “passive” components. Active time refers to tasks that require user interaction with the computer. Passive tasks only require the user to wait for the computer. Active processing time is determined by several factors. Suitable parameters for detecting cells need to be evaluated several times on ranges of images throughout the sequence. This takes at least a few minutes in all cases. Active track correction time is primarily determined by the number of cells in the microwell, but is also increased if a large number of cells are highly motile. Tracking errors are very rare when wells have few cells, or the cells are not highly motile. Specific details of our procedures are given in figures 8, 10 and section 4.3 in the main article.

For comparison, we are only able to obtain anecdotal evidence of the time required to produce data for [4]. The authors of that paper reported that lineage, cell division & death times were extracted using basic software tools (such as image editing, video playback, and spreadsheet programs). In [4], area and position information was only captured for a subset of the cells, due to the difficulty involved. For part of the cited work, semi-automatic segmentation and tracking was performed using Metamorph software. Metamorph uses a different approach to semi-automated segmentation and tracking, requiring that cell-tracks be manually initiated by clicking each cell. Metamorph then builds a model of the appearance of the cell and tries to identify it in future images using position and appearance information. Typically, Metamorph is then able to track cells for a small number of frames until the track is confused or lost.

For all methods of cell tracking the time required per microwell is highly variable, and primarily determined by the number of cells in the microwell (see tables S1, S2). For modelling lineage and cell events such as divisions and deaths without spatial data, in simple microwells with small populations (1 to 4 cells), there is some reduction in manual effort using our methods, but the overall time increases. For microwells with up to 8 cells there is some benefit to our methods. When microwells contain more than 8 cells, our methods appear to greatly reduce the amount of human labour and overall processing time while providing a much richer set of data.

When comparing the our methods to use of Metamorph and manual tracking (for lineage reconstruction), our methods are much faster.

Microwell max cells	Manual	Metamorph	TrackAssist (active)	TrackAssist (total)
1 to 4	15m	30m*(1 lineage)	6m	27m
5 to 8	2h	n/a	23m	45m
9 to 16	10h	n/a	38m	61m
17 to 32	24-32h	n/a	2h:27m	03h

Table S2. Anecdotal time requirements for cell tracking using existing software tools, compared to our methods. Note: "Manual" means use of basic software tools for recovery of lineage structure and death, division times only (i.e. the data lacks precise contour and cell position information). Note that manual times don't include time spent preparing videos and other files for this purpose, which can be laborious. "Metamorph" refers to use of semi-automated tracking with the Metamorph software package, without exact cell position or contour. This data was only available for 1 cell-lineage. "TrackAssist (active)" means time consumed actively interacting with our software. The user is not free to perform other tasks during these periods. "TrackAssist (total)" refers to total time elapsed using our methods, i.e. including time spent waiting for the software perform automatic processes. Max cells refers to the maximum number of living cells in the microwell at any time during the experiment.

4 Discrepancies in reproduction of population statistics

We found a slightly weaker correlation between founder area at first division and time to first division than Hawkins et al, although from a smaller number of founder cells analysed (figure S2). We also found a weaker correlation between founder area at first division and average division at death (figure S2). In Hawkins et al these are figures 4C and 4D respectively. We found no correlation between founder area at first division and time to second division (our figure S2, figure 4E in Hawkins et al).

Our figure S3 reproduces figure 5 panels A,B,C from Hawkins et al. These show correlations between division times of related cells. We found slightly weaker but still significant correlations between siblings, and between mothers and daughters. We found the same significant correlation between the division times of grandmothers and granddaughters.

Since we expected that tracking or other automation errors would reduce correlations we manually reviewed the 10 most extreme outliers in all these graphs. Our failure to observe the founder area and division time correlation was verified to be a property of the data and not an artefact of automated cell segmentation and tracking. However, our method of measuring cell area was based on fluorescence and as discussed below, may not always be a direct measure of cell size (see section 2.4 in the main article.).

In figure 2 (in the main article) we reproduce a number of cell size profiles. Although we observed the same "sawtooth" pattern of cell area during growth and division, in the final generation we observed some growth in some cells. This is discussed in greater detail below, with other new data.

5 Other observations from lymphocyte spatial data

We have made some additional observations and analysis of the intensive, multi-generational lymphocyte tracking and lineage data that have not been previously published.

5.1 Distribution of inter-cell distance over time and by fate

Without any fixed reference points in the microwells, we can only measure inter-cellular distances. There are also no clear clusters to measure distance from. Having found variations in motility between generations and fates, we wished to investigate any differences in inter-cellular distances over time. For example, do cells tend to spread out, or remain clustered? There is an inherent tendency for cells to be close to each other, when they are the progeny of a common ancestor. However, a Brownian motion model would

predict a gradual dispersal of cells until a uniform density was achieved. Figure S6 shows the distribution of inter-cellular distances observed over the course of our experiment.

1. *Over time, inter-cellular distances tend to increase but intra-population variance decreases*
   Figure S6 shows a slight reduction in the variance of inter-cellular distance within the cell populations, over time. However, the modal distance increases slightly in later generations. These results may not be meaningful. It is possible that uneven microwell floor surfaces and microscope stage motion could have influenced these characteristics.

   The reduction in variance does not conflict with the observation that cells slow down in later generations. Without a suitable motion model it is not possible to determine whether the increasing average distance is a real phenomenon or a random result. We expect that two competing processes - random motion (which would favour increasing separation) and cell division (which favours reducing separation) determine the overall relatively constant distributions of inter-cellular distance that we observed.

2. *No relationship between fate and inter-cellular distances*
   Despite our observation that cells fated to die tend to move more rapidly, we could observe no significant differences in inter-cellular separation between cells fated to die and cells fated to divide.

5.2 Correlations between ancestor cell size and descendant cell size, by fate

It is already known that lymphocyte proliferation usually results in successively smaller cells. We correlated the initial areas of parent cells with the initial areas of their progeny, for all progeny fates (figure S7). To reduce measurement noise we used a window of the first 36 size measurements (approximately 3 hours) of each cell. In both cases, as expected, daughters tended to be smaller at birth than mothers. The correlation observed supports expectations that generational reduction in size occurs consistently (i.e. a similar reduction in size occurs for all lineages and generations). We found the strongest correlation between mothers and daughters fated to die; a weaker correlation was found between mothers and daughters fated to divide.

5.3 Distributions of lymphocyte area over time and by fate

To present a detailed picture of lymphocyte area over time, generations and fates, we have produced histograms of lymphocyte area over time, for each generation and fate (figure S8). Some observations from these histograms include: Variance in the area of cells decreases with successive generations, particularly for cells fated to divide. Both the rate of cell growth and cell sizes achieved decrease over successive generations. There are clear differences in the distributions between different fates; cells fated to divide tend to grow, whereas cells fated to die tend to shrink.

Bi-Modal distribution of cell areas, for cells fated to die - We observe an unexpected bi-modal distribution of areas for cells fated to die in generations 3 and 5, but not in generations 4 and 6. This does not match any known biological phenomenon, leading us to question whether it could be a processing artefact. However, the same processing was applied to all generations (in fact, the segmentation algorithm is blind to cells' generation). The results might be explained if the cells had come from two related founding populations, but our data does not have any such grouping. We could not determine any systematic error that could account for this anomaly, leaving an open question for further research. Interestingly, the modal sizes of cells fated to die in generations 3 and 5 are slightly larger and smaller than the modal sizes of cells fated to divide in the same generations; i.e. both slightly larger than average and much smaller than average cells are more likely to die. While it was expected that smaller cells are more likely to die (see [4] for cell size relationship to chances of dying), a greater rate of death for larger lymphocytes was not anticipated. This effect is particularly distinct in generation 5.

5.4 Distributions of lymphocyte speed over time and by fate

Figure S9 shows histograms of cell motility (speed) over time, for each generation and for each fate separately. Two features of this data stand out. First, cells tend to slow down in later generations, regardless of fate. This was also shown in figure 4 in the main article. Second, cells fated to divide tend to move more slowly than cells fated to die. From the histograms, we can see that the modal speed of cells for both fates is very similar, but there is greater variance in cells fated to die. Since these experiments are *in vitro* it does not necessarily mean that the same effects would be observed *in vivo*, but the differences between fates are likely to reflect expressed characteristics of the cells.

Figures

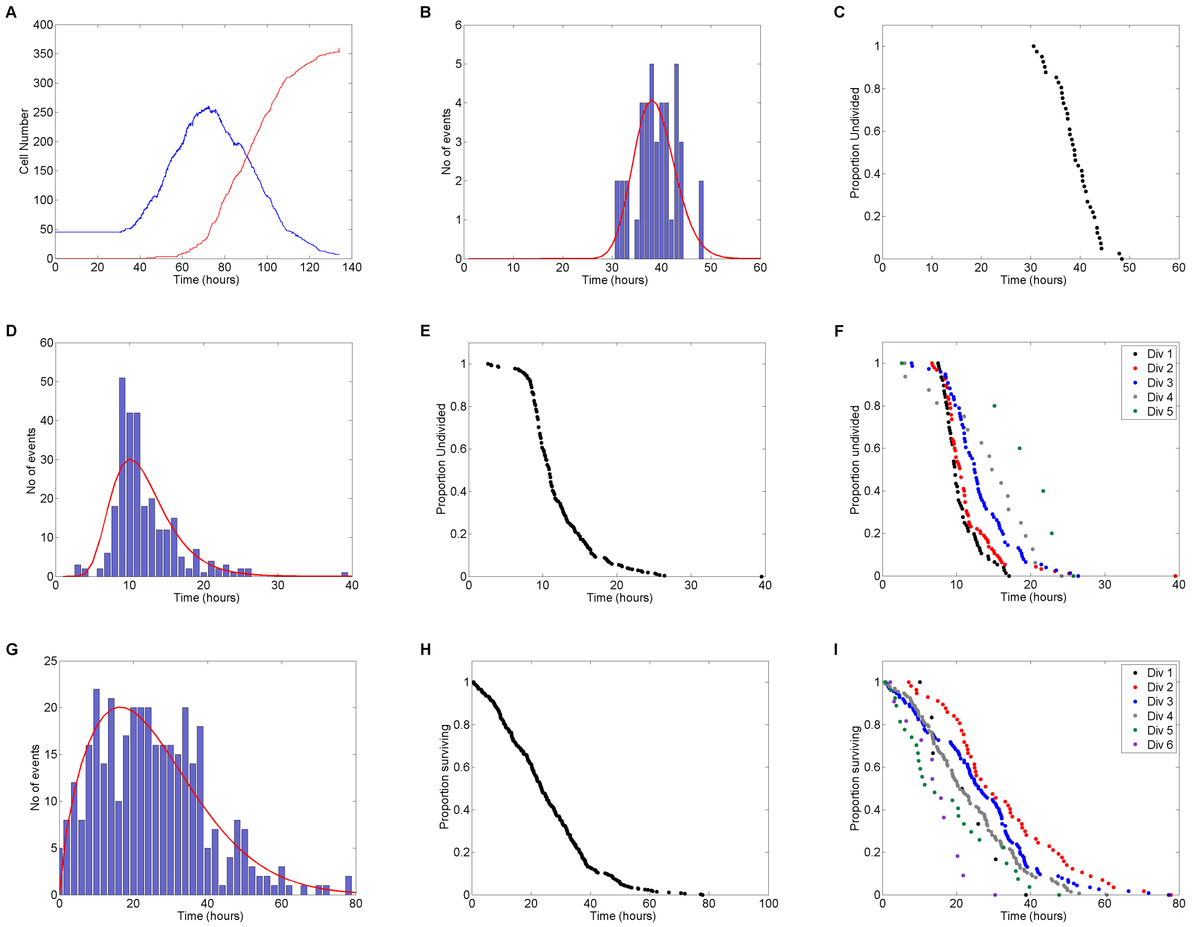

Figure S1. This figure is a reproduction of population statistics from figure 1 in Hawkins et al [4]. We have reproduced the figure using data from a different experiment, using the workflow and software described in this paper. We found good agreement between the original data and ours. Panel A: Total population of live (blue) and dead (red) cells over time. Panel B: Distribution of first division events over time, for cells extant at the start of the experiment. C: Showing the reduction of the undivided cell population over time. D,G: Distribution of subsequent division and death events respectively, over time. E,H: Alpha plots for remaining undivided and undead cells respectively, in all generations. F,I: Same data, but per generation.

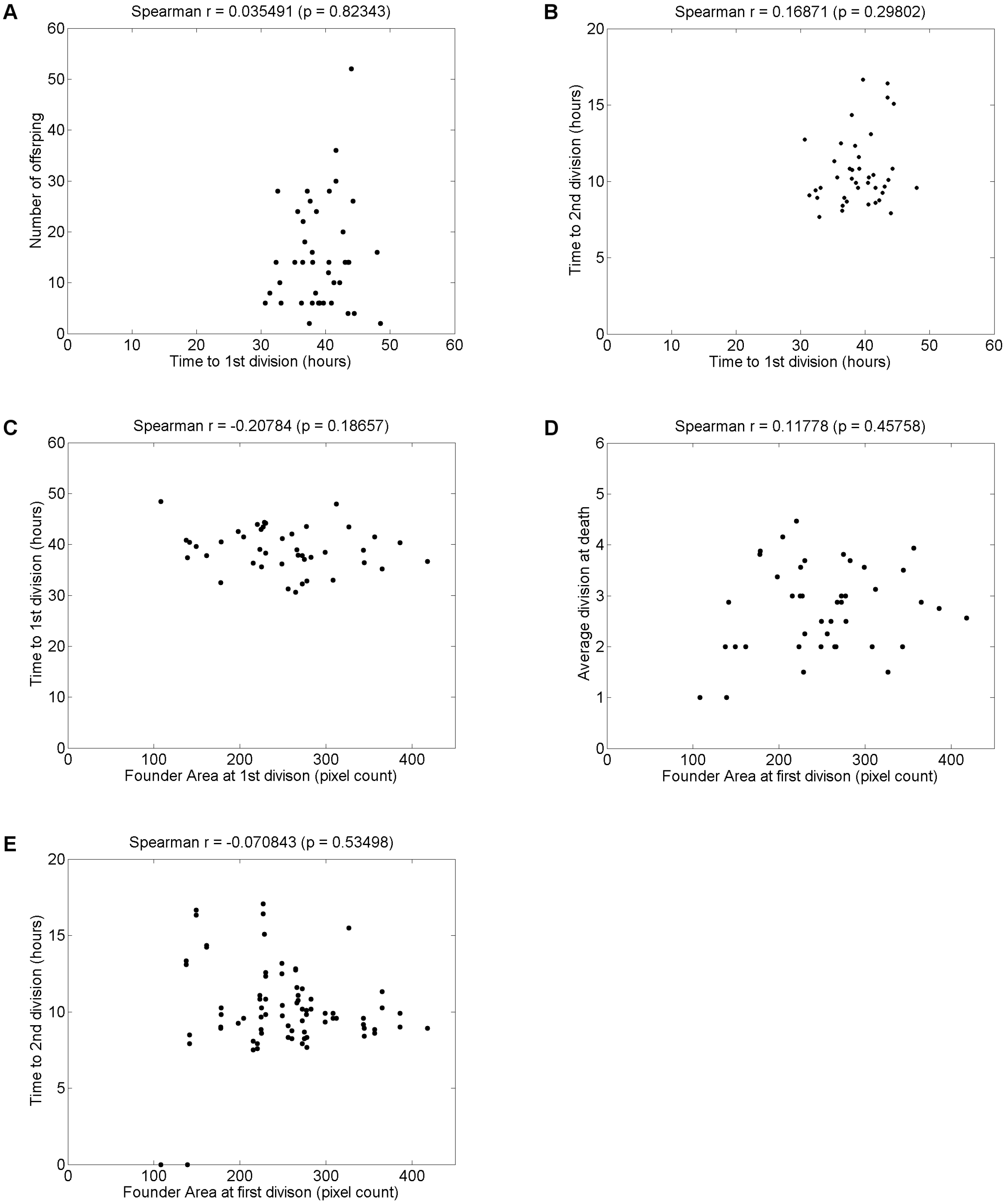

Figure S2. This figure is a reproduction of population statistics from figure 4 in [4]. The subfigures give Spearman's rank correlation coefficient and show correlations between A: number of offspring and time until first division. B: Time to first division and time until second division. C: Area at first division and time to first division. D: Area at first division and average progeny divisions prior to death. E: Area at first division and area at second division.

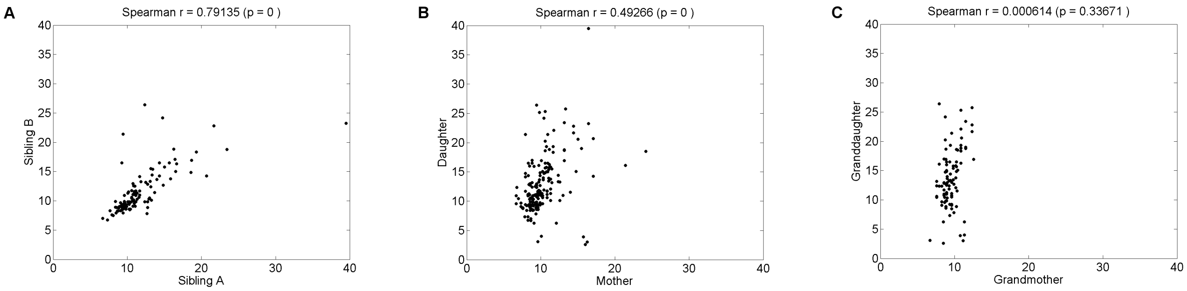

Figure S3. This figure is a reproduction of population statistics from figure 5 in [4]. These scatter graphs illustrate possible correlations between time-to-division for related cells. A: Showing relationship between sibling cells. B: Showing relationship between time to division for mother and daughter cells (1 generation gap). C: Between grandmother and granddaughter cells (2 generation gap).

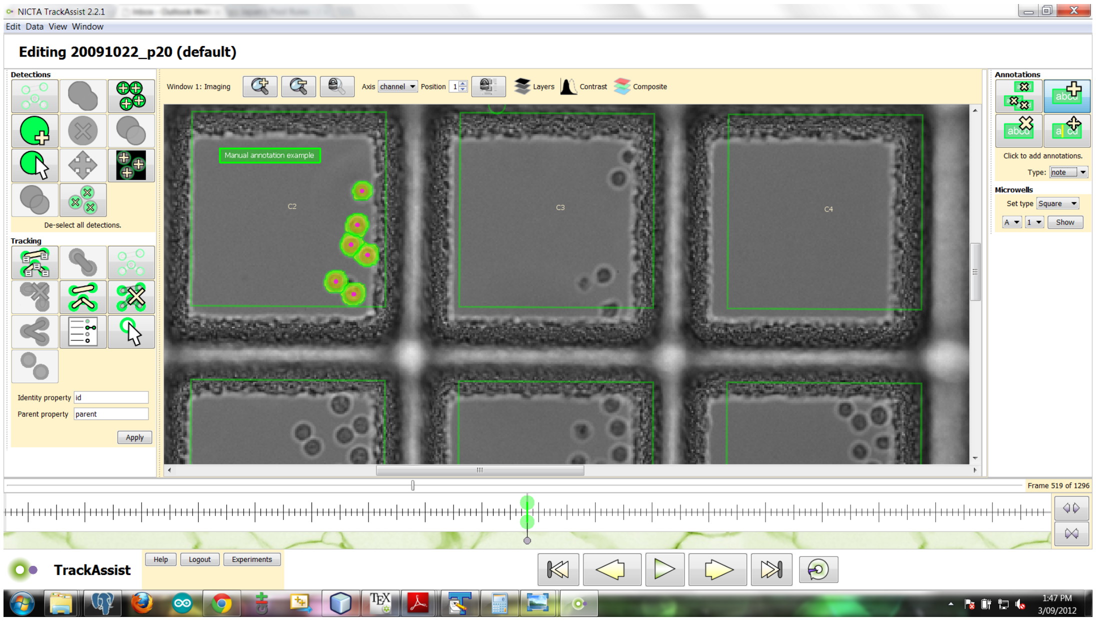

Figure S4. Graphical user interface of the TrackAssist software. This figure shows all track and detection editing tools (left), time navigation controls (bottom) and manual annotation tools (right). One annotation is shown in the image. A purple balloon in the time indicates frames that have annotations making them easy to find. The slider control allows gross adjustment of time and the ruler control gives fine navigation of time. Video-player style buttons control playback of the sequence or a window of time within the sequence. Controls at the top allow navigation of the various image channels and pan, zoom.

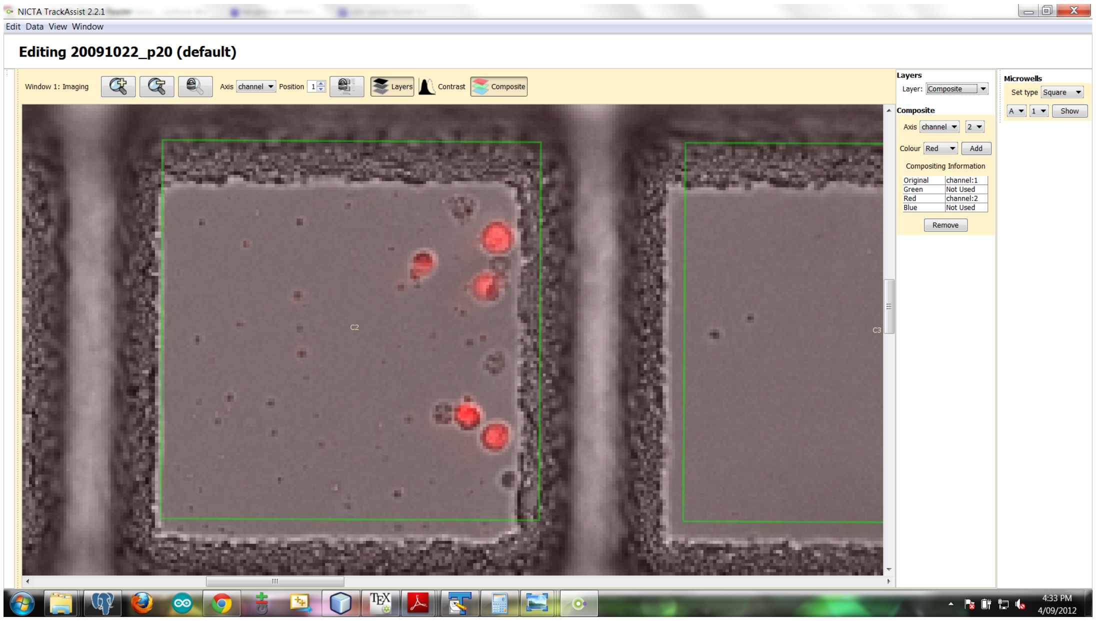

Figure S5. Graphical user interface of the TrackAssist software. This screenshot shows compositing of the transmission image and co-registered green filtered fluorescence image (shown here as a red channel overlay). Cells that are fluorescing appear to be red. Compositing assists users to interpret cell state by exploiting both structural information from the transmission image and cell-state cues from fluorescence images.

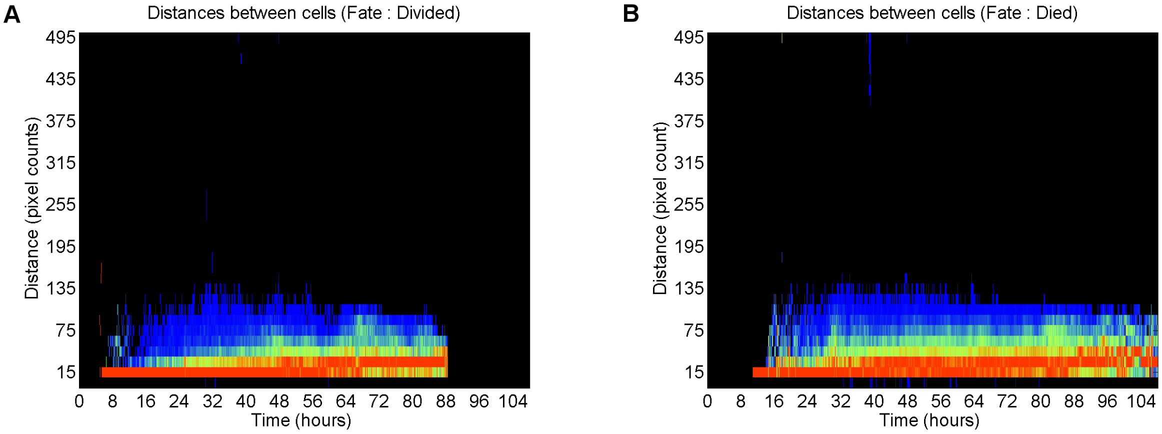

Figure S6. Inter-cellular distance distribution, by fate. The purpose of this presentation is to detect any patterns that may exist. Since there is no physical reference point in the highly artificial microwell environment, the only meaningful measurement that can be made for individual cells is inter-cellular distance. There are some extreme outliers, but most cells tend to remain close to each other. We concluded that there were no differences between cells fated to die and cells fated to divide.

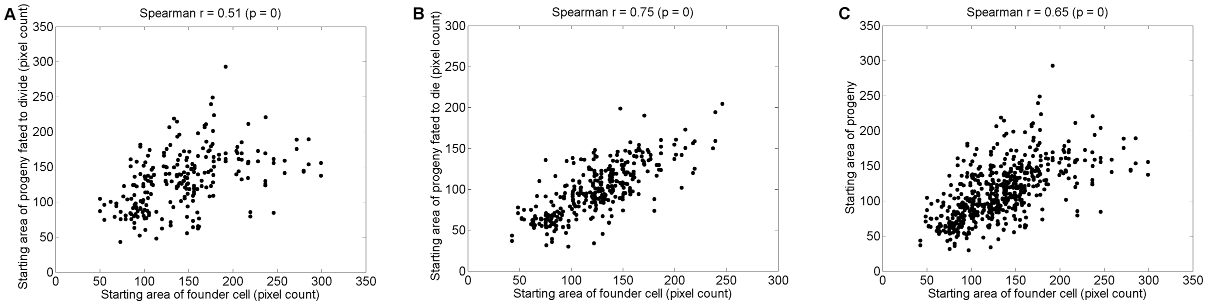

Figure S7. Correlations of cell size between lymphocytes and their descendants, by fate. In all cases there is a strong positive correlation between founder cell size and progeny cell size, regardless of fate. This graph was included because it was not available in Hawkins et al [4]. The correlation between mothers and daughters fated to die was strongest, rather than cells fated to divide. Since cells tend to grow in all generations but the last, this suggests that variations in mothers' growth reduces the correlation for that fate. For cells fated to die, there has typically been less growth in the penultimate generation. See figure 7 in the main article for rates of growth of cells by fate.

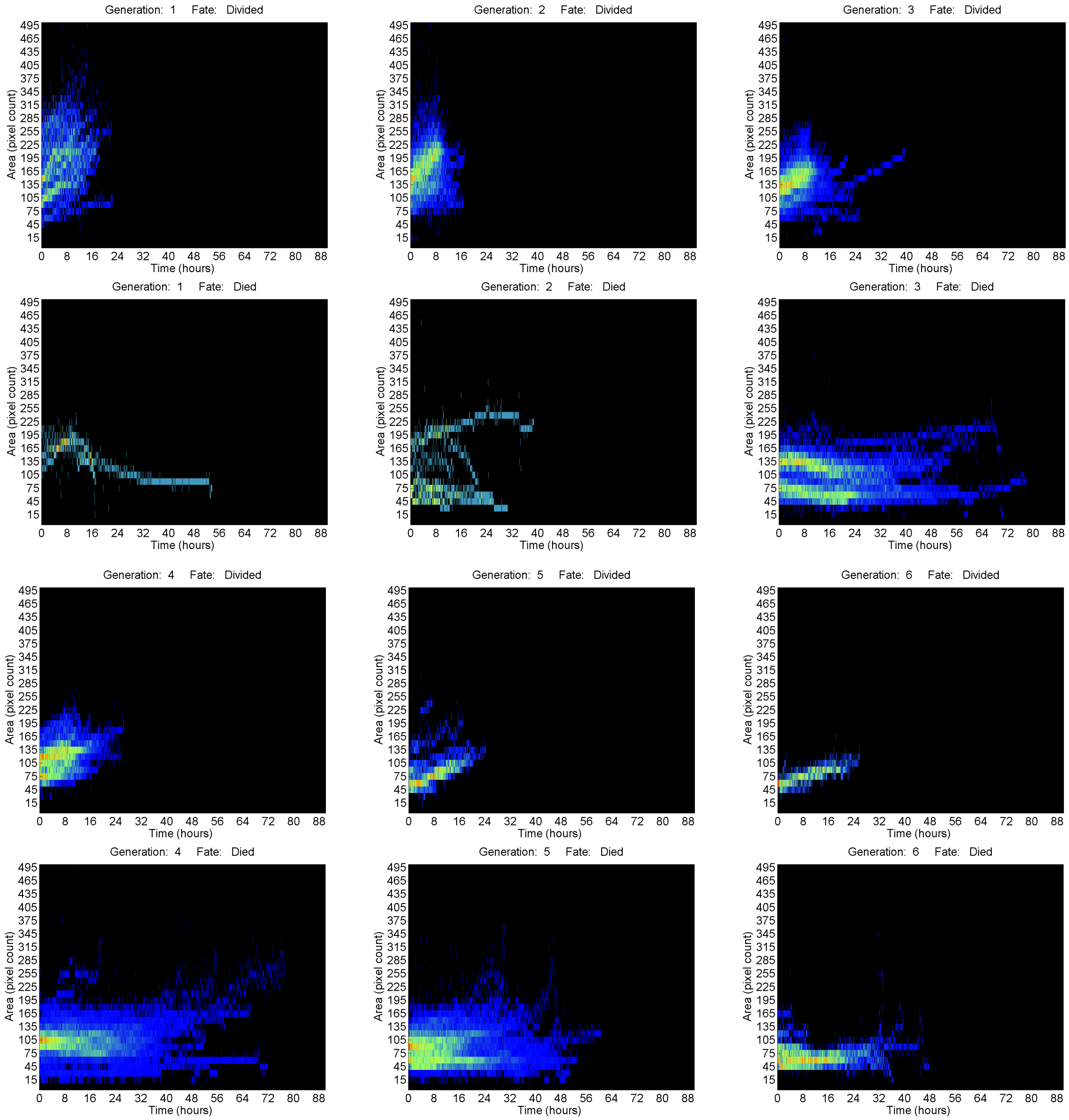

Figure S8. Histograms of cell area by generation and fate.

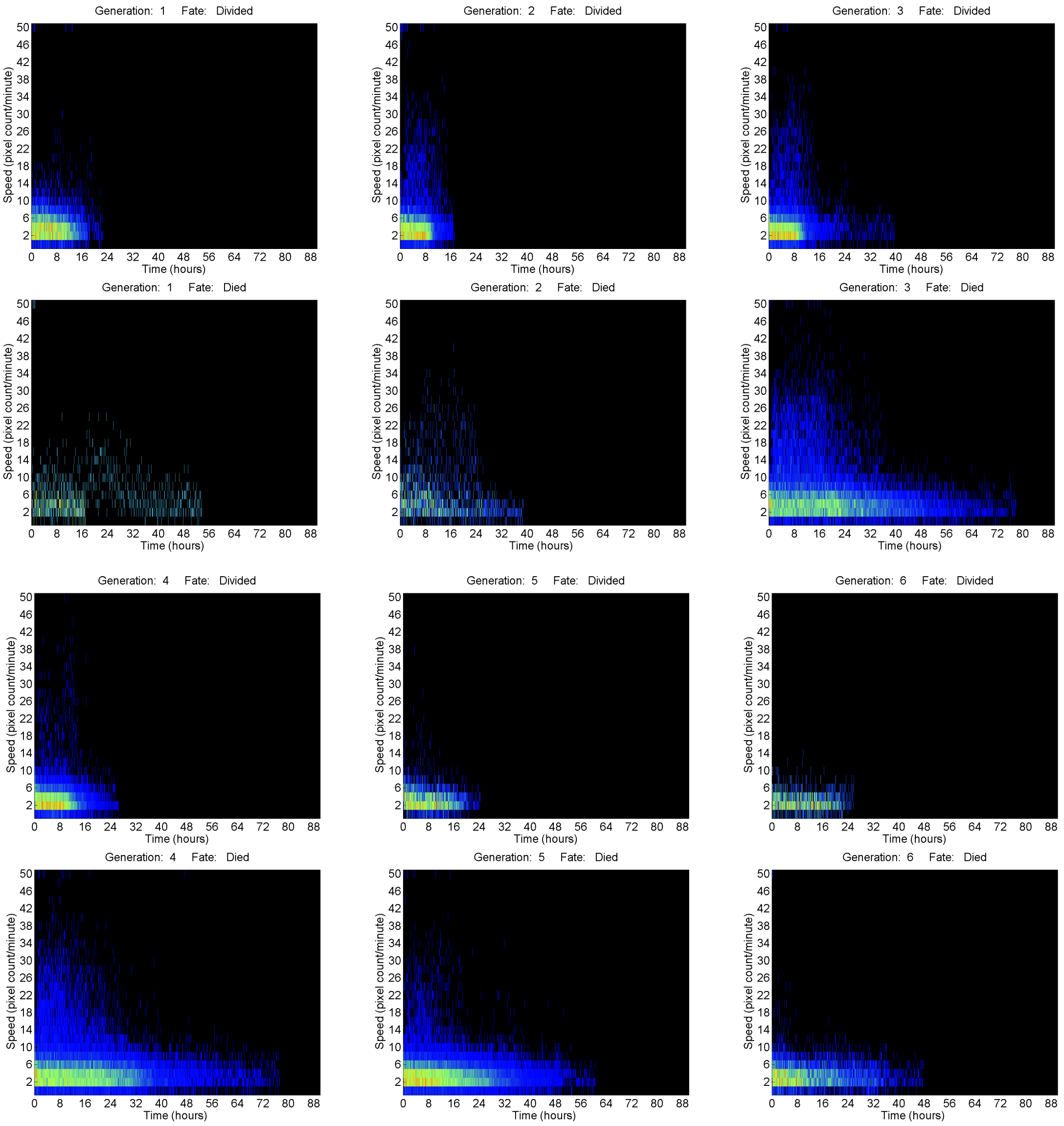

Figure S9. Histograms of cell speed by generation and fate.

References

1. Day D, Pham K, Ludford-Menting M, Oliaro J, Izon D, et al. (2009) A method for prolonged imaging of motile lymphocytes. Immunology and Cell Biology 87: 154-158.

2. Rasband W (1997-2012). Imagej. U.S. National Institutes of Health, Bethesda, Maryland, USA. URL http://imagej.nih.gov/ij. Accessed: 2010-09-30.

3. Gnu image manipulation program (gimp). URL http://www.gimp.org. Accessed: 2010-09-30.

4. Hawkins ED, Markham JF, McGuinness LP, Hodgkin PD (2009) A single-cell pedigree analysis of alternative stochastic lymphocyte fates. Proceedings of the National Academy of Sciences of the United States of America 106: 13457-13462.
